# Isocycloseram: A new active ingredient for leaf-cutting ants control

**DOI:** 10.1371/journal.pone.0300187

**Published:** 2024-05-09

**Authors:** Ronald Zanetti, Jessica J. Sanches, Andrea V. A. Wenzel, Khalid Haddi, Henrique Ferreira, Leandro V. Santos

**Affiliations:** 1 Departamento de Entomologia, Universidade Federal de Lavras, CEP 37200–900, Lavras, MG, Brasil; 2 Syngenta Proteção de Cultivos, CEP 04730–300, São Paulo, SP, Brasil; University of Carthage, TUNISIA

## Abstract

Leaf-cutting ants are the most important pests in several cropping systems in the Neotropics. Granulated baits containing active ingredients, considered hazardous by the Stockholm Convention, are the usual method to control these ants. Isocycloseram is a new insecticide molecule with high safety margin for mammals, but without registration for the ants in general. Thus, this study investigated the effectiveness of granulated baits with isocycloseram in leaf-cutting ants control under laboratory and field conditions. Initially, the mortality of *Atta sexdens* workers, fed with dehydrated citrus pulp paste containing different concentrations of isocycloseram was evaluated in the laboratory for 21 days, for toxicological classification. Subsequently, the loading, devolution, and incorporation of baits with different concentrations of isocycloseram and the mortality of *A*. *sexdens* colonies were evaluated in the laboratory. After that, the percentages of loading and devolution of baits, foraging activity, and colony mortality treated with 0.05, 0.1, 0.2, and 0.3% of isocycloseram were evaluated for the species *A*. *sexdens*, *A*. *laevigata*, and *Acromyrmex lundii* in field conditions. All concentrations of isocycloseram killed more than 15% of ants in 24 h and more than 90% in 21 days in the laboratory, being classified as a fast-acting and highly effective active ingredient. Baits with 0.001 to 0.03% of isocycloseram were highly loaded and exhibited low rate of devolution. The mortality of *A*. *sexdens* colony was higher at concentrations between 0.075 and 0.3%, in the laboratory. Baits containing isocycloseram at concentrations of 0.2 and 0.3% were highly loaded, presented low devolution rates, and were highly efficient in controlling *A*. *sexdens*, *A*. *laevigata*, and *A*. *lundii* in the field, at dosages of 6, 10, and 12 g/m² of nest. This is the first report of the use of isocycloseram against leaf-cutting ants, contributing to the development of efficient and toxicologically safer ant baits.

## Introduction

Leaf-cutting ants of the genera *Atta*, *Acromyrmex* and *Amoimyrmex* (Hymenoptera: Formicidae) are herbivores widely distributed throughout the Neotropics [1,2]. These ants harvest fresh plant material, primarily leaves, carry it into their nests, and use it as a growth medium to cultivate a symbiotic fungus (*Leucocoprinus gongylophorus*) on which they feed [3,4]. Leaf-cutting ants are known for their extensive underground nests sheltering millions of ants and reaching over 300 m^2^ of loose soil for *Atta*, and up to 20 m^2^, in the case of *Acromyrmex* [5] and *Amoimyrmex* [6].

Defoliation by leaf-cutting ants reduces plants’ photosynthetic rate and productivity, causing losses of billions of dollars annually to agricultural and forestry crops in South and Central America, where these ants are considered key pests [7,8]. Both broad leaf crops, such as cassava, coffee, cocoa, cotton, fruits, and soybean [7,9–11], as well as narrow leaf crops like pastures, and sugarcane [12,13], are frequently damaged by these insects. Additionally, forestry plantations including softwoods like pine [14–17] and hardwoods like eucalyptus [5,18,19] also suffer damage caused by leaf-cutting ants.

The control of leaf-cutting ants is predominantly achieved with toxic granulated baits [8], considered more practical and economical than other control methods [20]. However, few active ingredients are available and registered for use. Due to the biological and behavioral traits of leaf-cutting ants, an effective insecticide must not only be toxic to them, but also allow workers to forage, transport, and distribute the bait within their nests undeterred [21,22]. Fipronil and sulfluramid are the most used active ingredients in baits [4,8,23], but they are considered toxic to mammals and persistent in the environment by the Stockholm Convention and some forest certification schemes [24–27]. Alternative baits to sulfluramid and fipronil have been tested, but produced unsatisfactory results, motivating the development of new effective ingredients against leaf-cutting ants [26].

Isocycloseram is a new insecticide of the isoxazoline class synthesized in 2021 [28], classified in IRAC Group 30 [29]. It is a broad-spectrum insecticide and acaricide, with high safety margin for mammals, and toxic against Lepidoptera, Hemiptera, Coleoptera, Thysanoptera, and Diptera, without known cross-resistance [28,30,31]. Isocycloseram is an allosteric modulator of the GABA-controlled chloride channel acting at a different site than the previously known antagonists such as fiproles and cyclodienes. The prolonged activity of this compound at low concentrations indicates it may have utility as an active ingredient in ant bait formulations. In 2023, isocycloseram was marketed as Plinazolin^®^ technology of Syngenta Company.

Thus, this work aimed to (*i*) test and classify the potential use of isocycloseram in leaf-cutting ants’ control in laboratory; (*ii*) evaluate the effectiveness of granulated baits containing this active ingredient on *A*. *sexdens* colonies in laboratory and (*iii*) on *A*. *sexdens*, *A*. *laevigata*, and *A*. *lundii* colonies under field conditions.

## Materials and methods

### *In vitro* bioassay

*Atta sexdens* (Linnaeus) workers were taken from mature colonies (±12 years), maintained in laboratory conditions (24±2°C, 70±10% relative humidity, and 12 h-12 h L:D photoperiod). The colonies received fresh leaves of *Acalypha* spp. and *Hibiscus* spp., cornflakes, and water twice a day.

Baits with different isocycloseram concentrations (0.001, 0.01, 0.025, 0.05, 0.075, 0.1, 0.2, and 0.3%) were prepared by mixing corresponding quantities (weight/weight) of isocycloseram (98% purity) in 36 mL of 10% (weight/volume) sucrose solution. Then 12 g of dehydrated citrus pulp powder was mixed to obtain a homogeneous paste. Sulfluramid (96% purity) at 0.1% (weight/weight) was used as the positive control and water as the negative control (placebo). They were prepared and incorporated to the citrus pulp baits as described above.

Twenty medium-sized workers, with individual weights between 10 and 20 mg and head capsule widths between 2.0 and 2.8 mm, were placed in a 10 cm diameter Petri dish, lined with filter paper. Each dish received 2 g of the formulated paste of one of the treatments (one of isocycloseram concentrations or one of the controls), inside a plastic container (3 cm diameter x 0.3 cm height) (S1 Fig). Ants were allowed to feed on the formulated past for 24 h. After that, the formulated paste was replaced by a ± 2 g piece of an agar-based diet [32]. The diet was changed every 48 h, throughout the experimental period of 21 days [32]. A portion of fungal sponge of approximately 1 cm^3^, containing about 20 small gardener ants (∼1 mm) was added to each petri dish to feed the tested workers, throughout the experimental period [21] (S1 Fig). The gardener ants were introduced, because medium-size workers have difficulties in maintaining the fungus garden [33]. The Petri dishes remained in a BOD incubator for 21 days, with temperature set at 22 ± 1°C and total scotophase [21].

The experimental design consisted of 10 treatments: eight concentrations of isocycloseram, sulfluramid (0.1%), and a placebo (water); each with six replications (Petri dishes with 20 ants). The number of dead ants was evaluated on the 1^st^, 2^nd^, 3^rd^, 5^th^, 7^th^, 9^th^, 11^th^, 14^th^, 17^th^ and 21^st^ days after application of the treatments.

### Laboratory bioassay with colonies

Sixty-six 1.5-year-old *A*. *sexdens* colonies, with approximately 750 mL of fungus mass, were used for the bioassay. They were placed in 750 mL transparent plastic bottles, connected by transparent plastic tubes (2 cm in diameter and 10 cm in length) to two 750 mL bottles, arranged at 180°; one bottle served as a waste chamber and the other as the foraging chamber (S1 Fig). These colonies were maintained in the laboratory at 24 ± 2°C, RH of 70±10%, and 12-hour photophase, and received fresh leaves of *Acalypha* spp. and *Hibiscus* spp., cornflakes, and water twice a day.

Isocycloseram, with a purity of 98%, was diluted in degummed soybean oil (± 15%) and combined with dehydrated citrus pulp powder (± 85%). The mixture was then extruded through a pellet machine, resulting in granulated baits (pellets) measuring approximately 1.4 mm in diameter and 5.0 mm in length. These pellets contained varying concentrations of isocycloseram, specifically 0.001, 0.003, 0.025, 0.03, 0.05, 0.075, 0.1, 0.2, and 0.3% (w/w). The sulfluramid and placebo treatments were prepared in the same way, containing sulfluramid (96% purity) at 0.3% (weight/weight) and water, respectively. The experimental arrangement consisted of the nine concentrations of isocycloseram, sulfluramid and placebo, with six colonies (repetitions) each.

The colonies remained without food for 24 h before offering treatments. Thus, 0.5 g of the pellets was offered for 24 h. After that, the remaining pellets were removed, and the colonies received leaves of the *Acalypha* spp. and *Hibiscus* spp. daily (S1 Fig).

The percentage of baits loaded, devolution, and incorporated were evaluated on scales of 0, 25, 50, 75, and 100% [34]. Colonies engaging in cutting leaves, with or without antagonistic fungi, or experiencing mortality were assessed daily up to the third day. Subsequently, evaluations were performed every three days until the 42^nd^ day following the application of the baits.

### Field bioassays with colonies

Field bioassays were carried out in eucalyptus and pine plantations, with high nests infestation, in Minas Gerais (*Atta sexdens* (Linnaeus) ‐ 19°23´03 S, 45°07´07 W, 767 m; and *Atta laevigata* (Smith) - 18°07´23 S, 44°50´56 W, 691 m) and Santa Catarina (*Acromyrmex lundii* (Guérin-Méneville) *-* 27°46´46 S, 50°21´04 W, 805 m) states, Brazil. *Atta sexdens* and *A*. *laevigata* nests, with a size between 9 and 104 m², and *A*. *lundii* nests, between 1 and 2 m^2^ and that have never received insecticide, were selected (S2 Fig). The loose soil area was measured for all nests [35], and then they were distributed across treatments and doses in an effort to balance sizes evenly.

Baits of isocycloseram at concentrations of 0.05, 0.1, 0.2, and 0.3% (weight/weight), and placebo were prepared as in the previous subsection. The positive control was a commercial bait with sulfluramid at 0.3% (Dinagro-S^®^). Each concentration of isocycloseram was tested at doses of 6, 10, and 12 g of formulated pellets per square meter of loose soil for both *Atta* and *Acromyrmex* species nests. These doses are similar to the sulfluramid used in commercial products per square meter of nest, as they are related to the volume of the chambers and the number of ants in the nest [36]. In addition, using loose soil is relatively simple and practical for application in the field [37]. The sulfluramid (0.3%) and placebo were tested at a single dose of 10 g/m^2^. The experimental arrangement was randomized, with 14 treatments (four concentrations of isocycloseram with three doses per square meter each, one dose of sulfluramid, and one dose of placebo) and ten replications (nests) per treatment and species (*A*. *sexdens*, *A*. *laevigata*, and *A*. *lundii*).

The *Atta* nests’ loose soil area were determined [35], and the bait was applied 20 cm away from the active holes around the nests. The percentage of loading and devolution of baits was evaluated 24 and 48 h after application, using a 5% visual scale. Colonies’ activity was assessed based on the presence/absence of ants moving loose soil and cutting leaves on the 1^st^, 2^nd^, 3^rd^, 7^th^, 15^th^, 30^th^, 60^th^, 90^th^, 120^th^, and 150^th^ days after the bait application, in the case of the *Atta* species, and until the 90^th^ days after application, in the case of the *Acromyrmex* species. In the last evaluation, the colonies were excavated to confirm the colony death [38] (S1 Fig).

### Data analyses

In the *in vitro* bioassay, the number of dead ants per evaluation day was analysed using the Cox Proportion Hazards survival model. The proportion of dead ants 24 h and 21 days after application of the treatments were analysed by GLM generalized linear models, with binomial error distribution and Tukey’s test (p<0.05), and ants’ mortality percentages were used for the isocycloseram activity classification [21].

In the laboratory bioassay with colonies, the proportion of loading and devolution of the baits 24 and 48 h after the application of the treatments were analysed by GLM generalized linear models, with binomial error distribution and Tukey’s test (p<0.05). The proportion of colonies either showing leaves’ cutting activity or dead was analysed by a Cox Proportional Hazards survival and model contrast (p<0.05).

In the field bioassays, the proportion loading and devolution of baits for 24 and 48 h after application, and the proportion of the dead colonies at the end of the assessment period were analysed by GLM generalized linear models, with binomial error distribution and Tukey’s test (p<0.05). The number of active ant colonies, cutting leaves and digging the soil, was analysed by a Cox Proportional Hazards survival model and model contrast (p<0.05).

Analyses were performed using R software [39]. Survival analyses were performed using *survival* pack and *survminer* [40,41]. The quality of the GLM models was checked using the *DHARMa* packages [42], and averaging tests were performed using the *emmeans* and *multcomp* packages [43,44].

## Results

### *In vitro* bioassay

**Fig 1:**
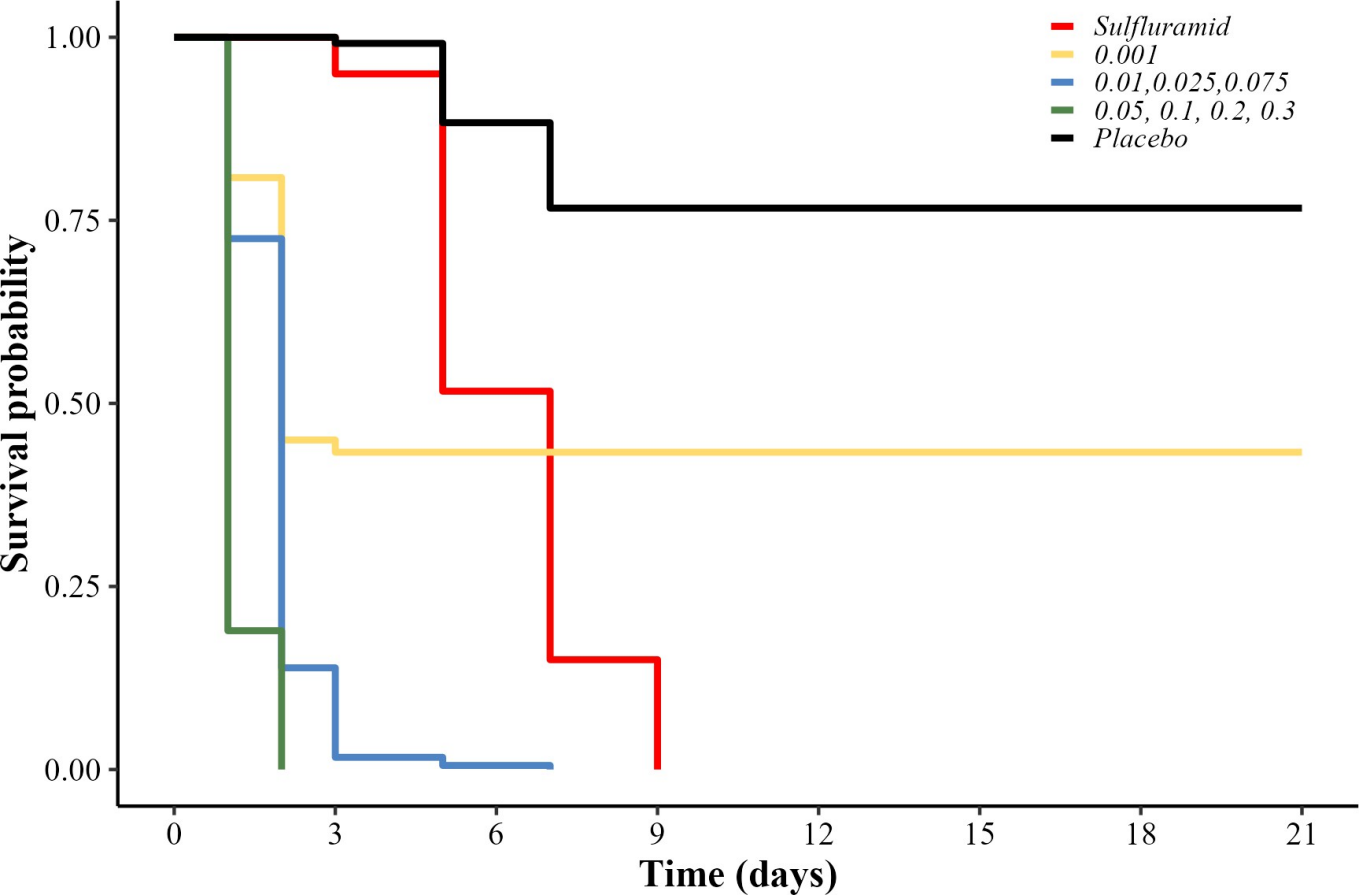
Survival probability of workers *in vitro*. Probability of survival of *Atta sexdens* workers fed *in vitro* with citrus pulp paste with isocycloseram at concentrations of 0.001, 0.01, 0.025, 0.05, 0.075, 0.1, 0.2, and 0.3%; sulfluramid at 0.1%; and water (placebo). Treatments represented by the same line (color) are statistically equal (p>0.05).

All the concentrations of both active ingredients (isocycloseram and sulfluramid), mixed with the citrus pulp paste, caused more mortality of *A*. *sexdens* workers than the placebo (Log-Rank: χ² = 324, df = 9, p<0.001, n = 1056). Besides the 0.001% concentration of isocycloseram, all the other concentrations of this active ingredient caused 100% ants’ mortality over the 21 days of evaluation, similar to the sulfluramid (0.1%). The ants treated with the placebo presented probability of survival over 80% (Fig 1).

**Fig 2:**
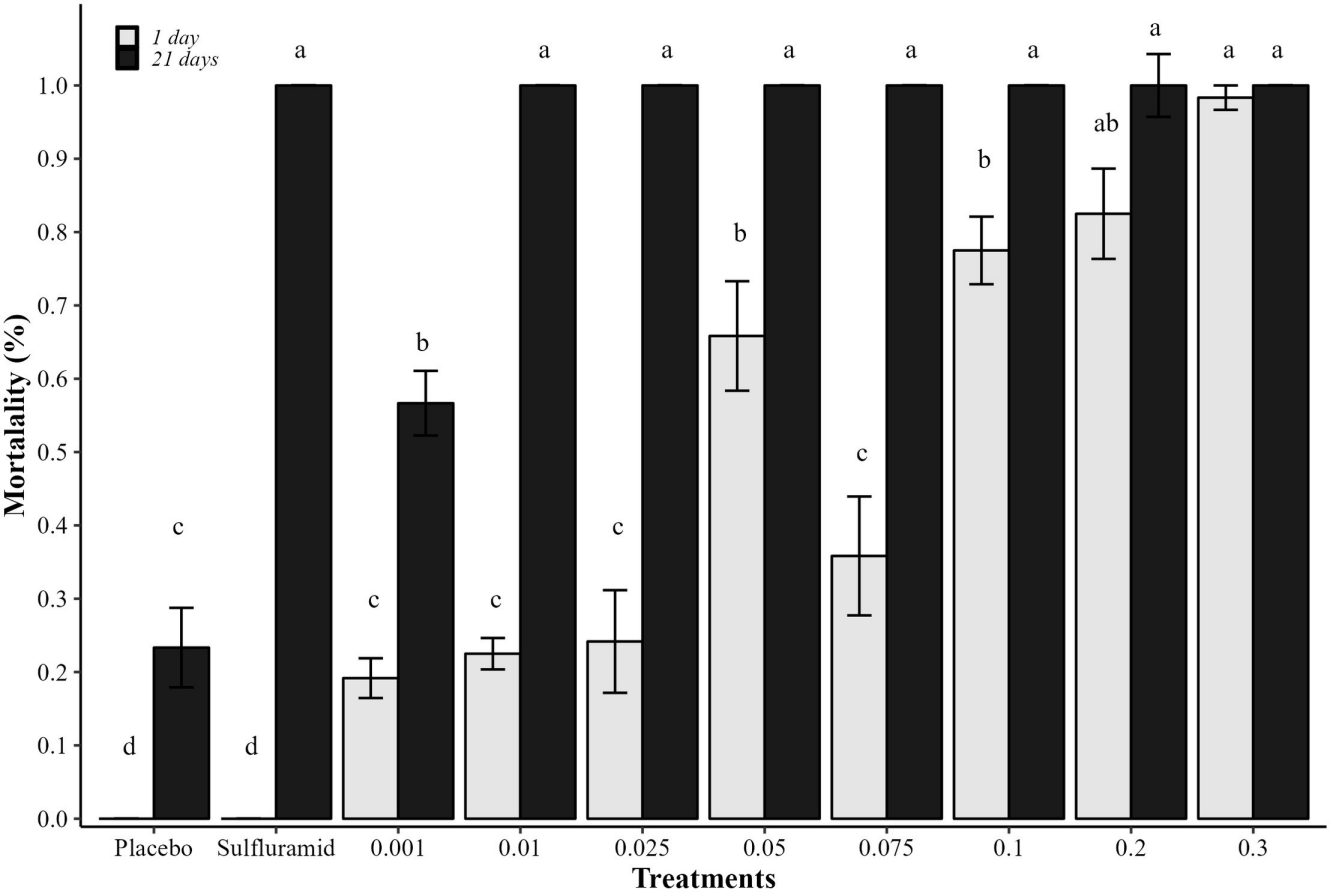
Mortality of workers *in vitro*. Mortality of *Atta sexdens* workers fed *in vitro* with citrus pulp paste containing: Isocycloseram at concentrations of 0.001, 0.01, 0.025, 0.05, 0.075, 0.1, 0.2, and 0.3%; sulfluramid at 0.1%; and water (placebo). Columns with the same letter for each evaluation period (1 or 21 days) do not differ from each other (Tukey post-hoc test; p>0.05). The bar in the columns indicates the standard errors of the means.

The percentage of dead ants 24 h and 21 days after application was different between treatments (χ²_(9;109)_ = 30.93, p<0.001). All isocycloseram concentrations killed more than 20% of the ants in the first 24 h after application of the treatments, characterizing the active ingredient as “fast-acting” (mortality > 15% in 24 h) (Fig 2). The mean lethal time (LT_50_) of isocycloseram was 1 to 2 days, while LT_50_ of the positive control was 7 days. In the comparison of accumulated mortality after 21 days, isocycloseram concentrations were as lethal as the sulfluramid (0.1%), with 100% mortality, except for 0.001%, with mortality lower than 60%. Then, the isocycloseram was characterized as an active ingredient for fast-acting ant control (mortality < 15% at 24 hours and> 90% with 21 days after application) [21] (Fig 2).

### Laboratory bioassay with colonies

**Fig 3:**
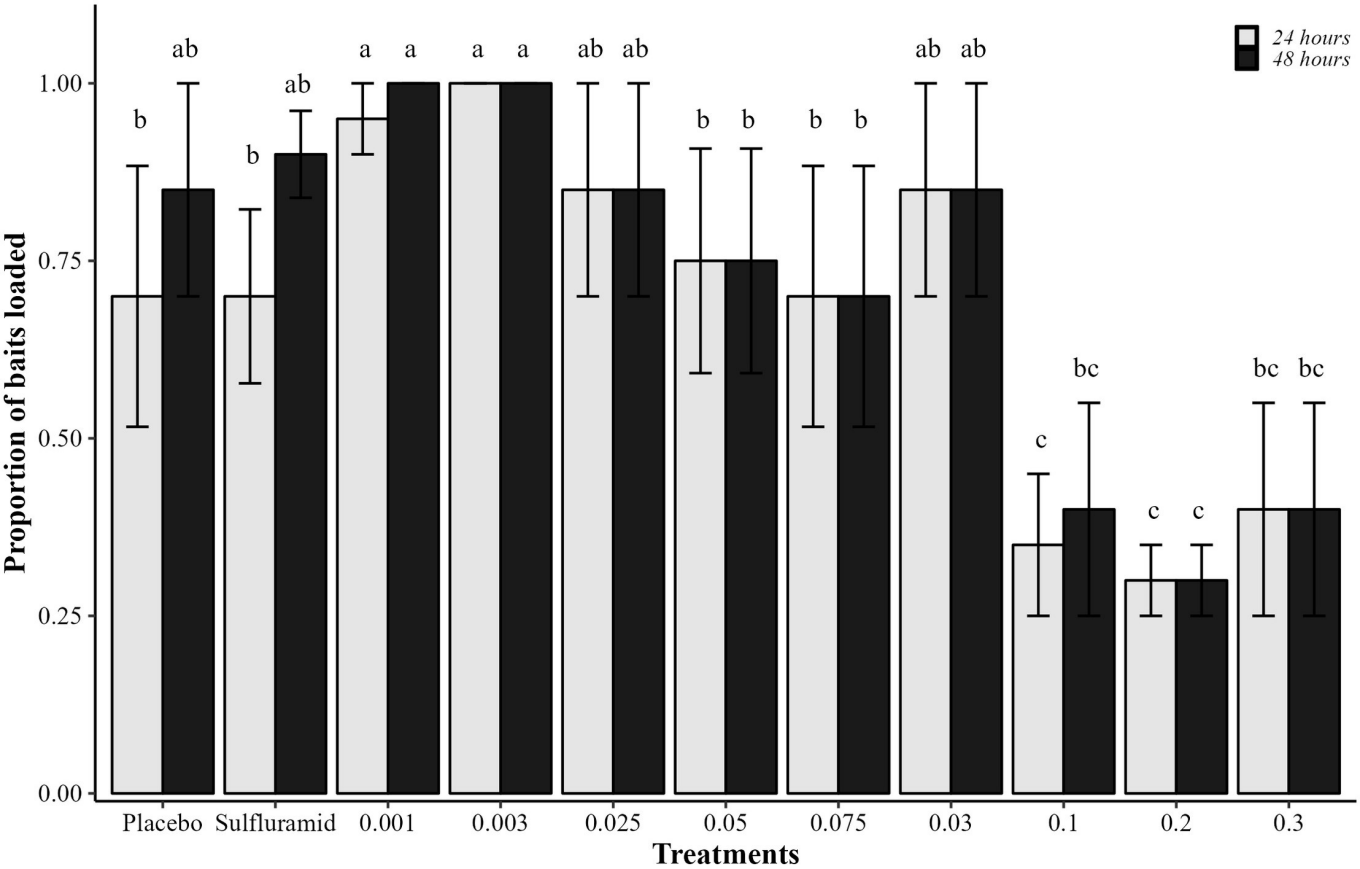
Proportions of baits loaded. Proportions of baits containing: Isocycloseram at concentrations of 0.001, 0.003, 0.025, 0.03, 0.05, 0.075, 0.1, 0.2, and 0.3%; sulfluramid at 0.3%; and water (placebo) loaded by *Atta sexdens*. Columns with the same letter, in each assessment period (24 or 48 h), do not differ from each other (Tukey post-hoc test; p<0.05). Bars on top of the columns show the standard errors of the means.

The percentage of bait loaded varied between treatments (χ²_(10.44)_ = 14.716, p<0.001) (Fig 3). The pellets were highly loaded by the ants in the first 24 h after application, except in the concentrations of 0.1, 0.2, and 0.3% of isocycloseram. However, bait loading at 48 h increased at concentrations of 0.1 and 0.3% after 48 h, equalling to placebo and sulfluramid. No devolution of bait was observed in any treatment, and the incorporation rate was 94.1 ± 7.7%, with no difference between treatments (χ²_(10.44)_ = 10.26, p>0.05).

**Fig 4:**
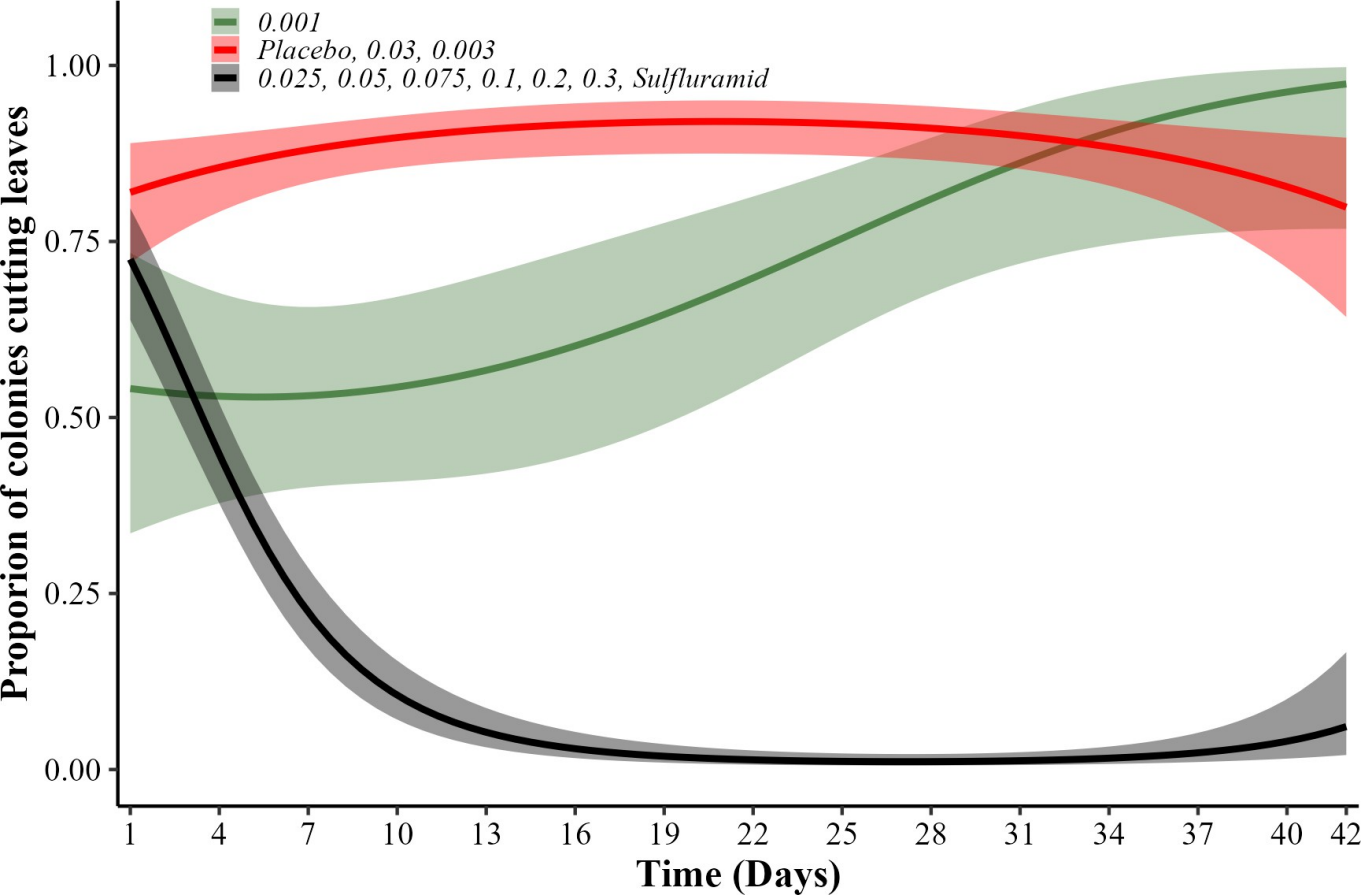
Proportion of colonies cutting leaves in laboratory. *Atta sexdens* colonies cutting leaves, after application of baits containing: Isocycloseram at concentrations of 0.001, 0.003, 0.025, 0.03, 0.05, 0.075, 0.1, 0.2, and 0.3%; sulfluramid at 0.3%; and water (placebo). Treatments represented by the same line (color) are statistically equal (p>0.05).

The proportion of colonies cutting leaves differed between treatments (χ²_(10.813)_ = 459.07, p<0.001). Leaves cutting rates decreased in the colonies that received the sulfluramid and isocycloseram at concentrations of 0.025%, 0.005%, 0.075%, 0.1%, 0,2% and 0.3%, unlike the other treatments that presented high rate of leaves cutting activity (Fig 4).

**Fig 5:**
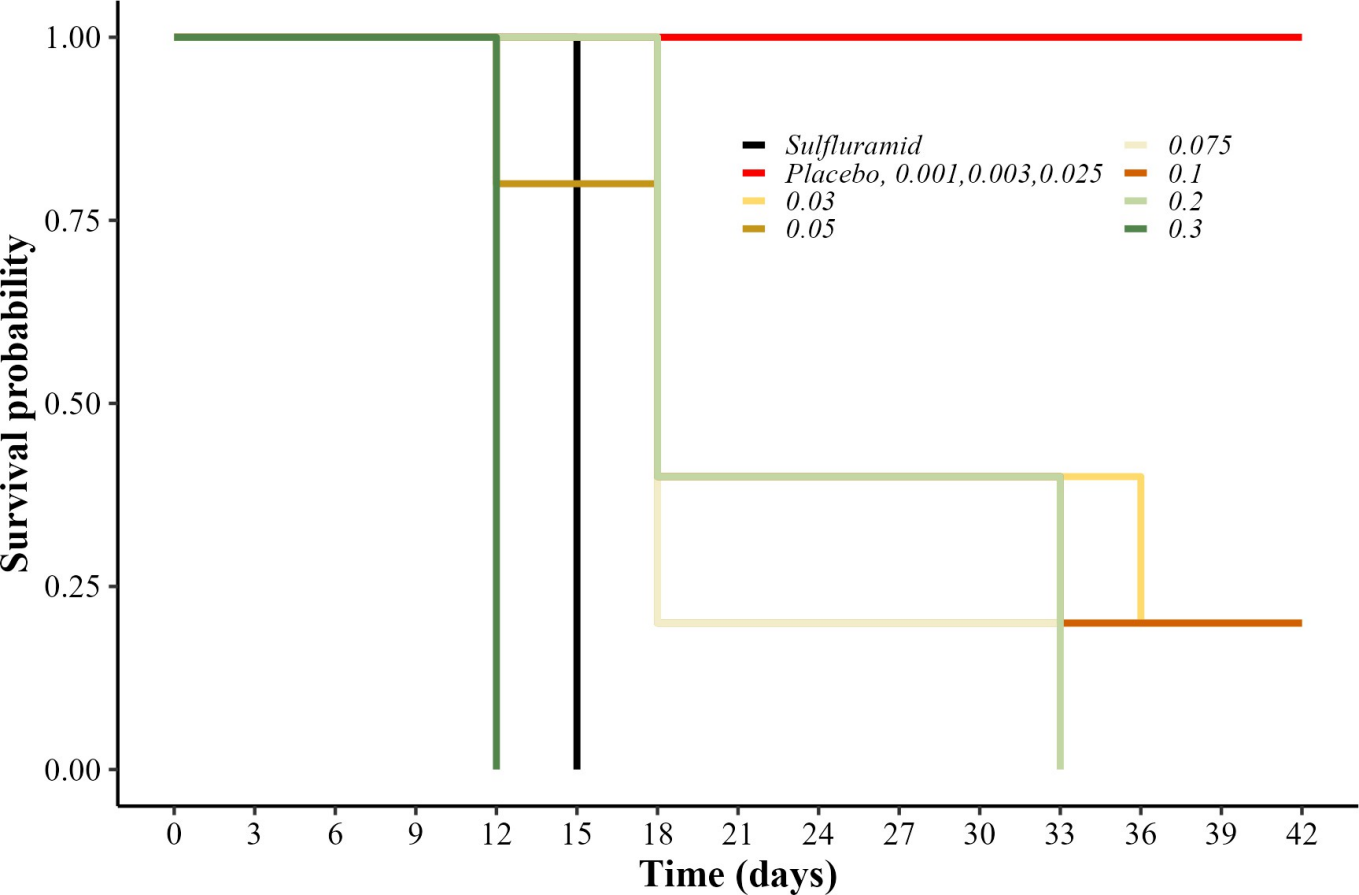
Survival probability of colonies in laboratory. Survival of *Atta sexdens* colonies treated with baits containing: Isocycloseram at concentrations of 0.001, 0.003, 0.025, 0.03, 0.05, 0.075, 0.1, 0.2, and 0.3%; sulfluramid at 0.3%; and water (placebo). Log-Rank indicated difference between treatments. Treatments represented by the same line (color) are statistically equal (p>0.05).

The percentage of colony mortality differed between treatments (χ² = 324, df = 9, Log-Rank p<0.001, n = 220). Similar to the sulfluramid (0.3%), isocycloseram concentrations above 0.03% caused high mortality (>80%). Isocycloseram at 0.3% had the lowest LT_50_ value with 12 days, followed by the positive control with 15 days and isocycloseram at 0.05, 0.075, 0.1, and 0.2% with 18 days. In all dead colonies, the presence and contamination by antagonistic fungi, such as *Escovopsis* sp. was observed (S1 Fig). The others isocycloseram concentration equal or lower than 0.025% were similar to the placebo and did not cause any colony mortality (Fig 5).

### Field bioassays with colonies

There was no difference in the rate of loaded baits by ants between treatments and doses for any of the species (*A*. *sexdens ‐ *χ²_(5.134)_ = 1.425, p>0.05; *A*. *laevigata ‐ *χ²_(5.134)_ = 3.260, p>0.05; and *A*. *lundii* ‐ χ²_(5.124)_ = 7.724, p>0.05), with a mean of 94.0 ± 9.8% of pellets transported to the nests. The devolution of bait was low (<1.5%) for all species, treatments, and doses, with no difference between treatments.

**Fig 6:**
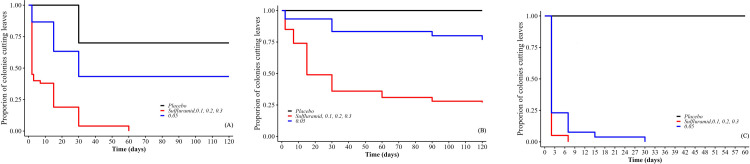
Proportion of colonies cutting leaves in the field. Proportion of colonies of *Atta sexdens* (A), *Atta laevigata* (B), and *Acromyrmex lundii* (C) cutting leaves, after the application of baits containing: Isocycloseram at concentrations of 0.05, 0.1, 0.2, and 0.3%, with a dosage of 6, 10, and 12 g/m^2^ of nest area; sulfluramid at 0.3%, at a dosage of 10 g/m^2^ of nest area; and water (placebo), at a dosage of 10 g/m^2^ of nest area. For *A*. *lundii*, the dosages of baits used were in g/nest. Treatments represented by the same line (color) are statistically equal (p>0.05).

The proportion of colonies cutting leaves at the end of the observation period depended of the ant species (Fig 6). Significant differences between treatments (*A*. *sexdens*: χ² = 8.03, p<0.001; *A*. *laevigata*: χ² = 5.551, p<0.001; and *A*. *lundii*: χ² = 8.82, p<0.001), but not between doses (*A*. *sexdens*: ‐ χ², = 8.98, p>0.05; *A*. *laevigata*: χ² = 0.909, p>0.05; and *A*. *lundii*: χ² = 8.98, p >0.05) were found for all species (Fig 6).

All colonies of *A*. *sexdens* treated with isocycloseram at concentrations 0.1, 0.2, and 0.3%, at all dosages, stopped the foraging activity no later than 60 days after the bait application (100%), similar to the sulfuramid (0.3%). Colonies treated with the placebo and 0.05% of isocycloseram differed from the other treatments and 40% of them keeping the foraging activity until the end of the observation period (Fig 6A).

Seventy per cent of colonies of *A*. *laevigata* that received pellets with 0.1, 0.2 and 0.3% isocycloseram stopped foraging no later than 90 days after application, as well as with the sulfluramid (0.3%), in all dosages, unlike those that were offered pellets with isocycloseram at concentrations of 0.05% (Fig 6B). All isocycloseram concentrations and dosages stopped foraging of *A*. *lundii* colonies up to 30 days after application, showing an identical effect as the positive control (Fig 6C). The median lethal time (LT_50_) to stop foraging of the isocycloseram treatments was three days for *A*. *sexdens* and *A*. *lundii* and 15 days for *A*. *laevigata*, similar to the sulfluramid, except for the 0.05% concentration (Fig 6).

**Fig 7:**
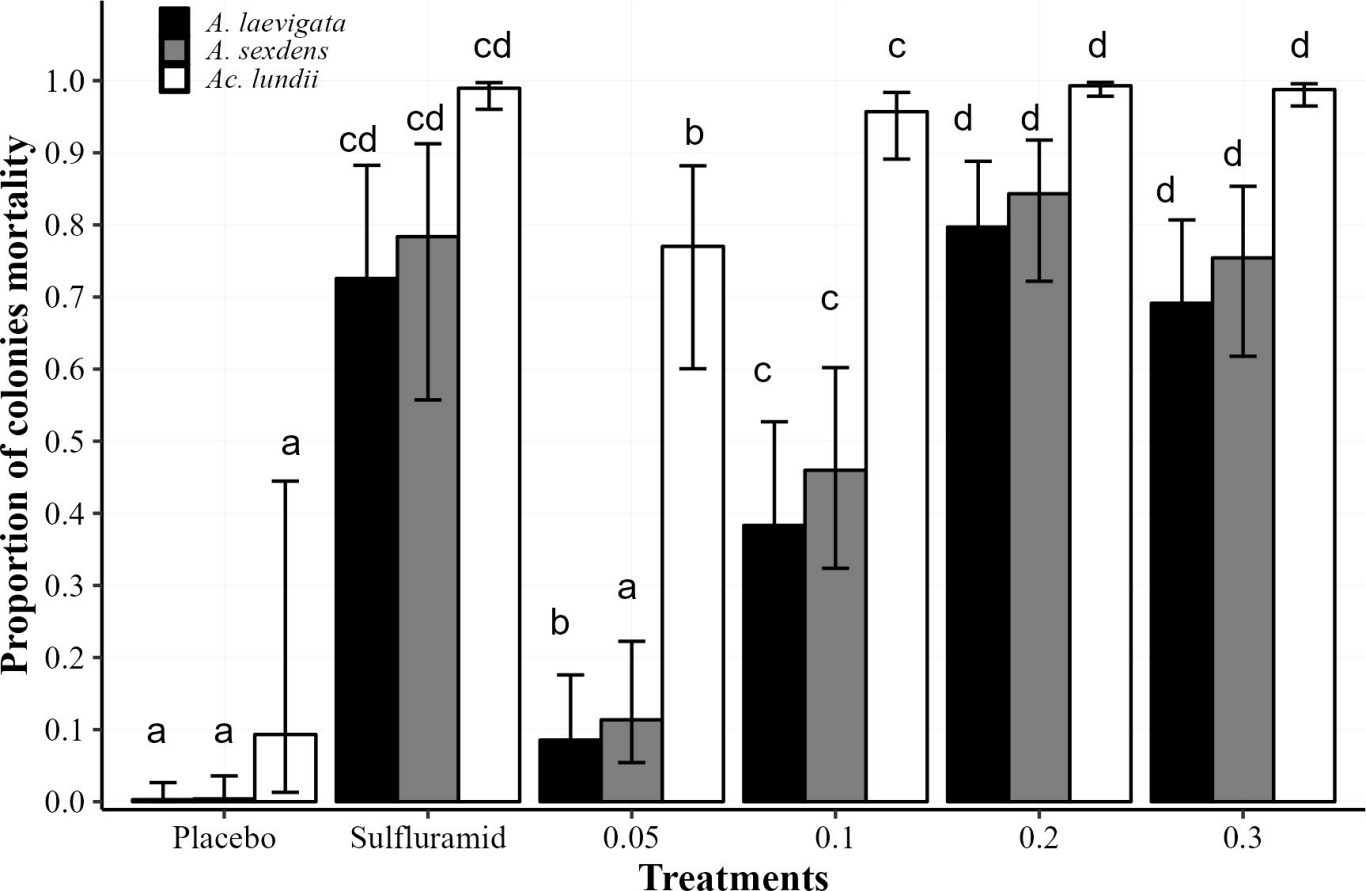
Proportion of colonies mortality in the field. Proportion of mortality of *Atta sexdens* and *Atta laevigata* colonies after 150 days; and *Acromyrmex lundii*, after 90 days, of baits application containing: Isocycloseram, at concentrations 0.05, 0.1, 0.2, and 0.3%, with a dosage of 6, 10, and 12 g/m^2^ of nest area; sulfluramid at 0.3% with a dosage of 10g/m^2^ of nest area; and water (placebo), with a dosage of 10g/m^2^ of nest area. For *A*. *lundii*, the dosages of baits used were in g/nest. Columns with the same letter, for each species, do not differ from each other (Tukey post-hoc test; p>0.05). Bars on the top of columns indicate the standard errors of the means.

Colonies’ mortality varied between treatments (χ²_(5,406)_ = 60.691; p<0001), ant species (χ²_(2,406)_ = 59.144; p<0.001), but not between doses of each treatment (χ²_(8,406)_ = 60.691; p> 0.05) (Fig 7). The isocycloseram at concentrations of 0.2 and 0.3%, at all dosages, and the sulfuramid (0.3%) killed an average of 77.5% of *A*. *sexdens* and *A*. *laevigata* colonies. The mortality of *A*. *lundii* colonies was greater than 90% for all isocycloseram concentrations and dosages, similar to sulfluramid.

## Discussion

### *In vitro* bioassay

The mortality of *A*. *sexdens* workers exposed to citrus pulp containing isocycloseram was higher than 15% in 24 h and greater than 90% after 21 days, except for the 0.001% concentration (<60%), which classified isocycloseram in class II ‐ “quick and efficient action” [21]. This indication differs from the main active ingredients of formicide baits used for leaf-cutting ants’ control, such as fipronil and sulfluramid, classified in classes III and IV, respectively–“delayed and efficient action” [21]. Leaf-cutting ants are extremely selective insects and can detect substances that can be toxic to the colony [45,46]. In this context, an ideal active ingredient for formicide bait should act by ingestion and have a delayed action. Its toxicity should not be detected by the ants before the loading, distribution, and incorporation in the colony’s fungus gardens [46,47]. Thus, formicide baits should have a slow effect on worker mortality, as has been observed for baits with sulfluramid [48,49] and reported in this essay. However, the exact range of delayed action has not been determined for any leaf-cutting ant species, which may mislead compounds’ attribution to toxicity classes [21]. Therefore, some active ingredients considered “fast-acting” may be suitable for baits. High loading and almost no devolution of baits in the laboratory and field tests using colonies reinforces the suitably of isocycloseram as formicide bait.

A suitable bait should be carried and incorporated into the fungus garden before ant mortality begins. The incorporation of baits into colonies of *A*. *sexdens* occurs relatively quickly in the laboratory [50], starting approximately 6 h after the supply of pellets and ending about 18 h later [4]. However, *in vitro* tests classify the potential of an active ingredient in a formicide bait after 24 h. Therefore, some active ingredients like thiamethoxam–class II [21,51], which were previously labeled as “fast-acting” and “unsuitable” for baits, may be actually effective if causing mortality in a shorter timeframe like less than 24 h allows enough baits to be distributed in the colony before worker ants die. Despite the high mortality of worker ants caused by isocycloseram in the first 24 h in the *in vitro* assay, the results obtained after 48 h are similar to those for *A*. *sexdens* when exposed to fipronil (>80%) at concentrations of 0.01 and 0.1% [52]. Isocycloseram and fipronil act on the GABA system, but in different ways [28], affecting the central nervous system, unlike sulfluramid, which acts in the oxidative phosphorylation process (at the mitochondrial level) and affects ATP production [53]. Furthermore, the pellets were loaded and incorporated into the fungus garden without causing mortality of *A*. *sexdens* workers during this process, in tests with colonies in the laboratory. The mortality rate obtained at the end of the 21^st^ day of evaluation, being similar to the one caused by sulfluramid, suggests that isocycloseram is adequate for use in formicide baits, as proved in the field bioassays.

### Laboratory bioassay with colonies

The ants loaded most of the baits in the first 24 h after application, except for pellets with isocycloseram at concentrations of 0.1, 0.2, and 0.3%, with less than 50% loaded by ants. However, bait loading increased at concentrations of 0.1 and 0.3% after 48 h, equalling to placebo and sulfluramid. Delays in loading baits into the field could increase their exposure to non-target species. Additionally, baits could absorb more moisture and receive more sunlight and high temperatures for long periods, reducing loading and effectiveness [4]. However, the baits applied in the field bioassay, even at the highest concentrations, were highly loaded, reducing these potential risks. Ideally, an effective toxic bait should not alter foraging behaviour or cause mortality during baits loading and incorporation, increasing the distribution of the bait in the colony [2,46], as occurred with isocycloseram. Generally, citrus pulp granulated-based baits are highly attractive to leaf-cutting ants. However, the high concentrations of isocycloseram may have been perceived as toxic by ants before full loading, as was observed for other ingredients, such as chlorpyrifos [54]. It is important to mention that the number of baits with isocycloseram applied (0.5 g) may have been high for a colony of 750 mL of fungus containing few workers, accelerating ant contamination and interruption of foraging at these higher concentrations. The tested baits with other concentrations of isocycloseram were quite attractive, as the worker-ants loading activity of these pellets was similar to what was reported for some extremely attractive formicide baits, such as sulfluramid [8] and dodecachlor [54]. Thus, using bait with this ingredient in lower concentrations and highly attractive to the ants, but at higher doses per square meter of loose soil, could increase the amount of isocycloseram in nests in the field, maintaining high loading and high efficiency.

The null devolution of baits with isocycloseram reinforces it potential as formicide. Leaf-cutting ants have a high ability to recognize substances that may harm the symbiotic fungus or contaminate the workers during foraging [55,56] and may return the product or isolate the contaminated chambers [22,46]. Even at high concentrations, where loading was lower than expected, there was low devolution of bait and high incorporation into the fungus garden, similar to dodechlorine [54], sulfluramid [48], chlorpyrifos [57], and fipronil [58], which have already been used or tested. As there was low devolution, workers could carry more bait after the 48 h established in the test protocol [21,38]. The low devolution and high loading of isocycloseram baits in the field, even at the highest concentration and dose per square meter of nest, indicate that the product is well accepted by ants and it is suitable as a formicide [59,60].

The sort period of stop the foraging activity by colonies treated with isocycloseram (except in concentrations below 0.03%) and with the sulfluramid (0.3%) is in line with effects previously reported for *A*. *sexdens* and *A*. *capiguara* Gonçalves colonies [49]. The great advantage of using chemical products to control insect pests is the possibility of immediate or rapid cessation of defoliation that may affect crop productivity. The defoliation caused by leaf-cutting ants is very severe and can kill young plants and reduce the growth of older ones [5]. Defoliation is most intense in the first 30 days after planting [61] and can be irreversible, as the seedlings are fragile and easily damaged [62,63], justifying the need to immediately stopping the foraging activity, without compromising loading and control effectiveness, as occurred with isocycloseram in laboratory and field bioassays.

Isocycloseram concentrations above 0.03% caused the highest percentage of colony mortality, similar to the sulfluramid. The observed presence of antagonistic fungi in dead colonies, such as *Escovopsis* sp., suggests that the colony died because the garden workers died and antagonistic fungi took over, weakening and causing a lethal infection to the colony [64–67], as observed with *Acromyrmex subterraneus* (Forel) colonies after offering baits with fipronil (0.003%) [58]. The health of a leaf-cutting ant colony and its mutualistic fungus depends on the gardeners’ constant care in removing fungi and other antagonistic microorganisms [67].

### Field bioassays with colonies

The high percentage of bait loaded (around 94%) in the first 48 h after application indicates the attractiveness and good acceptance of citrus pulp baits with isocycloseram by *Atta* and *Acromyrmex* workers. These results are similar to those obtained for *A*. *sexdens*, *A*. *laevigata*, and *Acromyrmex laticeps nigrosetosus* (Forel) treated with sulfluramid bait [49,57,68–70]. Nests of *A*. *sexdens* and *A*. *laevigata* treated with isocycloseram, at concentrations of 0.2 and 0.3%, showed the lowest foraging rates, similar to the sulfluramid. For *A*. *lundii*, isocycloseram at a concentration of 0.1% was as effective as the other ones. Then, the concentrations of 0.2 and 0.3% of isocycloseram, at dosages of 6, 10, and 12 g/m² of loose soil, proved to be the most suitable as a formicide, as they showed excellent results for both species. The development of chemical products for leaf-cutting ant control should consider efficiency for different ant genera and species [71], as they cut a wide range of plants [72,73] and occur in the same area and the same time [8], increasing the importance of this bait to leaf-cutting ant control in the different crops and agroecosystems. The higher bait loading in the field than in the laboratory were likely due to the larger colonies sizes on the field with a greater number of workers capable of carrying the bait. Worker size polymorphism increases with colony age, with large and older colonies having, on average, larger workers [74], and greater foraging capacity than smaller, younger ones [75].

The mean lethal time (LT_50_) until foraging stopped was three days for *A*. *sexdens* and *A*. *lundii* and 15 days for *A*. *laevigata*. Overall, colonies treated with isocycloseram needed similar times to that of sulfluramid to stop foraging, which is a great advantage in controlling leaf-cutting ants, as it allows to reduces the damage caused by the intense ants’ herbivory, as discussed previously. In addition, the use of bait that allow a quick cessation of foraging, in the early stage of cultivation can help reducing the use of more toxic methods, such as powdered insecticides [4], as it promotes the rapid damage control, preventing the defoliation and death of young plants.

## Conclusions

Granulated baits containing isocycloseram at concentrations of 0.2 and 0.3% are highly loaded, presented low devolution rates, and highly efficient in controlling colonies of *A*. *sexdens*, *A*. *laevigata*, and *A*. *lundii*, at dosages of 6, 10, and 12 g/m² of loose soil, being adequate for the management of leaf-cutting ants. This is the first report of the effectiveness of isocycloseram against ants, specifically leaf-cutting ants, contributing to the development of efficient and environmentally safe ant baits.

## Supporting information

S1 Fig Details of laboratory and field bioassays. Plate with formicide paste (A), diet and fungus (B) used in the bioassay *in vitro*; colony with leaves (C) and bait (D) used in laboratory bioassay; overview of colonies (E) and fungus contamination (F) in laboratory bioassay; and excavation of nests (G) and chambers of the dead *Atta* (H) and *Acromyrmex* (I) colonies in field bioassays.
(TIF)

S2 Fig Location of nests in the field. Location of *Atta sexdens* (A), *Atta laevigata* (B), and *Acromyrmex lundii* (C) nests in field bioassays.
(TIF)

S1 File Database. Raw data of *in vitro* bioassay, laboratory bioassay, and field bioassays with colonies of leaf-cutting ants.
(PDF)
