# Supplementary figures and images for: Isocycloseram: A new active ingredient for leaf-cutting ants control
Source: PLoS One. 2024 May 9;19(5):e0300187. doi: 10.1371/journal.pone.0300187 (PMC11081378; doi:10.1371/journal.pone.0300187)

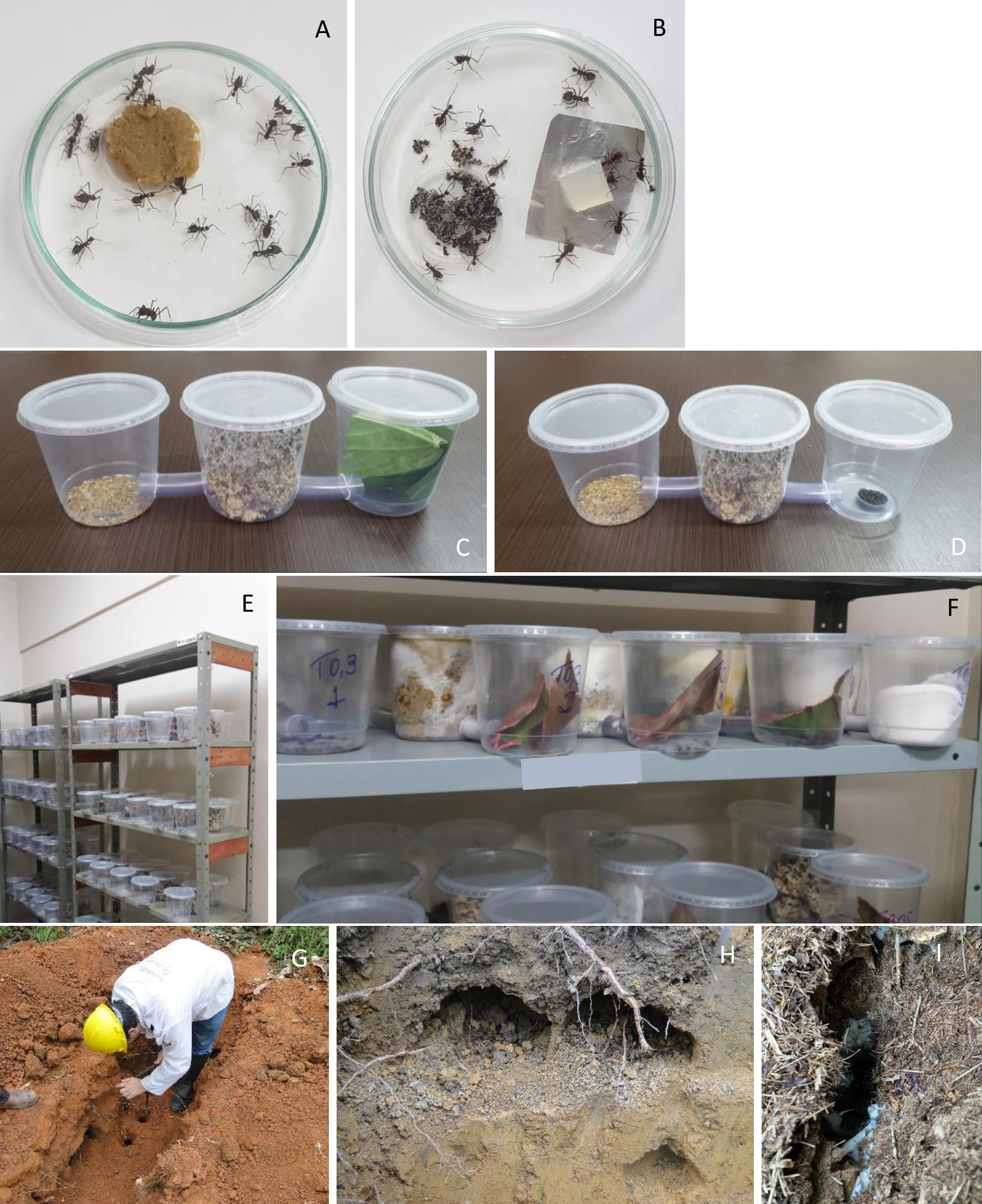

Supplement: S1 Fig — Plate with formicide paste (A), diet and fungus (B) used in the bioassay in vitro; colony with leaves (C) and bait (D) used in laboratory bioassay; overview of colonies (E) and fungus contamination (F) in laboratory bioassay; and excavation of nests (G) and chambers of the dead Atta (H) and Acromyrmex (I) colonies in field bioassays. (TIF) [file pone.0300187.s001.tif]

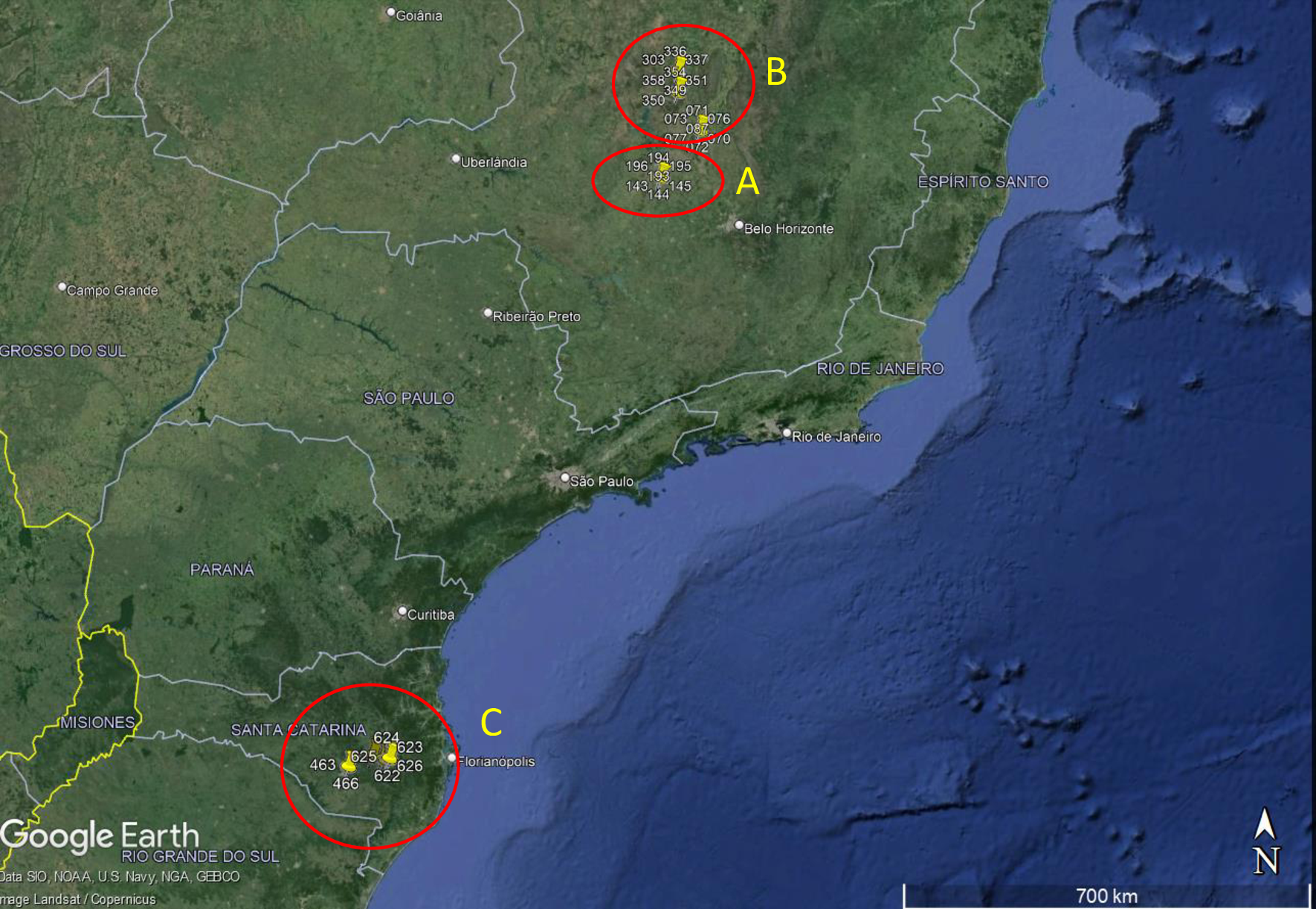

Supplement: S2 Fig — Location of Atta sexdens (A), Atta laevigata (B), and Acromyrmex lundii (C) nests in field bioassays. (TIF) [file pone.0300187.s002.tif]
