# Supplementary material for: Isocycloseram: A new active ingredient for leaf-cutting ants control
Source: PLoS One. 2024 May 9;19(5):e0300187. doi: 10.1371/journal.pone.0300187 (PMC11081378; doi:10.1371/journal.pone.0300187)
Supplement: S1 File — Raw data of in vitro bioassay, laboratory bioassay, and field bioassays with colonies of leaf-cutting ants. (PDF) [file pone.0300187.s003.pdf]

Database

Bioassay in vitro - Fig1 and Fig2

treatment	time	mortality	censor
control	3	1	1
control	3	2	1
control	3	3	1
control	3	4	1
control	3	5	1
control	3	6	1
control	5	1	1
control	5	2	1
control	5	3	1
control	5	4	1
control	5	5	1
control	5	6	1
control	5	7	1
control	5	8	1
control	5	9	1
control	5	10	1
control	5	11	1
control	5	12	1
control	5	13	1
control	5	14	1
control	5	15	1
control	5	16	1
control	5	17	1
control	5	18	1
control	5	19	1
control	5	20	1
control	5	21	1
control	5	22	1
control	5	23	1
control	5	24	1
control	5	25	1
control	5	26	1
control	5	27	1
control	5	28	1
control	5	29	1
control	5	30	1
control	5	31	1
control	5	32	1
control	5	33	1
control	5	34	1

control	5	35	1
control	5	36	1
control	5	37	1
control	5	38	1
control	5	39	1
control	5	40	1
control	5	41	1
control	5	42	1
control	5	43	1
control	5	44	1
control	5	45	1
control	5	46	1
control	5	47	1
control	5	48	1
control	5	49	1
control	5	50	1
control	5	51	1
control	5	52	1
control	7	1	1
control	7	2	1
control	7	3	1
control	7	4	1
control	7	5	1
control	7	6	1
control	7	7	1
control	7	8	1
control	7	9	1
control	7	10	1
control	7	11	1
control	7	12	1
control	7	13	1
control	7	14	1
control	7	15	1
control	7	16	1
control	7	17	1
control	7	18	1
control	7	19	1
control	7	20	1
control	7	21	1
control	7	22	1
control	7	23	1
control	7	24	1
control	7	25	1
control	7	26	1
control	7	27	1

control	7	28	1
control	7	29	1
control	7	30	1
control	7	31	1
control	7	32	1
control	7	33	1
control	7	34	1
control	7	35	1
control	7	36	1
control	7	37	1
control	7	38	1
control	7	39	1
control	7	40	1
control	7	41	1
control	7	42	1
control	7	43	1
control	7	44	1
control	9	1	1
control	9	2	1
control	9	3	1
control	9	4	1
control	9	5	1
control	9	6	1
control	9	7	1
control	9	8	1
control	9	9	1
control	9	10	1
control	9	11	1
control	9	12	1
control	9	13	1
control	9	14	1
control	9	15	1
control	9	16	1
control	9	17	1
control	9	18	1
I-0,001	1	1	1
I-0,001	1	2	1
I-0,001	1	3	1
I-0,001	1	4	1
I-0,001	1	5	1
I-0,001	1	6	1
I-0,001	1	7	1
I-0,001	1	8	1
I-0,001	1	9	1
I-0,001	1	10	1

I-0,001	1	11	1
I-0,001	1	12	1
I-0,001	1	13	1
I-0,001	1	14	1
I-0,001	1	15	1
I-0,001	1	16	1
I-0,001	1	17	1
I-0,001	1	18	1
I-0,001	1	19	1
I-0,001	1	20	1
I-0,001	1	21	1
I-0,001	1	22	1
I-0,001	1	23	1
I-0,001	2	1	1
I-0,001	2	2	1
I-0,001	2	3	1
I-0,001	2	4	1
I-0,001	2	5	1
I-0,001	2	6	1
I-0,001	2	7	1
I-0,001	2	8	1
I-0,001	2	9	1
I-0,001	2	10	1
I-0,001	2	11	1
I-0,001	2	12	1
I-0,001	2	13	1
I-0,001	2	14	1
I-0,001	2	15	1
I-0,001	2	16	1
I-0,001	2	17	1
I-0,001	2	18	1
I-0,001	2	19	1
I-0,001	2	20	1
I-0,001	2	21	1
I-0,001	2	22	1
I-0,001	2	23	1
I-0,001	2	24	1
I-0,001	2	25	1
I-0,001	2	26	1
I-0,001	2	27	1
I-0,001	2	28	1
I-0,001	2	29	1
I-0,001	2	30	1
I-0,001	2	31	1
I-0,001	2	32	1

I-0,001	2	33	1
I-0,001	2	34	1
I-0,001	2	35	1
I-0,001	2	36	1
I-0,001	2	37	1
I-0,001	2	38	1
I-0,001	2	39	1
I-0,001	2	40	1
I-0,001	2	41	1
I-0,001	2	42	1
I-0,001	2	43	1
I-0,001	3	1	1
I-0,001	3	2	1
I-0,001	21	1	0
I-0,001	21	2	0
I-0,001	21	3	0
I-0,001	21	4	0
I-0,001	21	5	0
I-0,001	21	6	0
I-0,001	21	7	0
I-0,001	21	8	0
I-0,001	21	9	0
I-0,001	21	10	0
I-0,001	21	11	0
I-0,001	21	12	0
I-0,001	21	13	0
I-0,001	21	14	0
I-0,001	21	15	0
I-0,001	21	16	0
I-0,001	21	17	0
I-0,001	21	18	0
I-0,001	21	19	0
I-0,001	21	20	0
I-0,001	21	21	0
I-0,001	21	22	0
I-0,001	21	23	0
I-0,001	21	24	0
I-0,001	21	25	0
I-0,001	21	26	0
I-0,001	21	27	0
I-0,001	21	28	0
I-0,001	21	29	0
I-0,001	21	30	0
I-0,001	21	31	0
I-0,001	21	32	0

I-0,001	21	33	0
I-0,001	21	34	0
I-0,001	21	35	0
I-0,001	21	36	0
I-0,001	21	37	0
I-0,001	21	38	0
I-0,001	21	39	0
I-0,001	21	40	0
I-0,001	21	41	0
I-0,001	21	42	0
I-0,001	21	43	0
I-0,001	21	44	0
I-0,001	21	45	0
I-0,001	21	46	0
I-0,001	21	47	0
I-0,001	21	48	0
I-0,001	21	49	0
I-0,001	21	50	0
I-0,001	21	51	0
I-0,001	21	52	0
I-0,01	1	1	1
I-0,01	1	2	1
I-0,01	1	3	1
I-0,01	1	4	1
I-0,01	1	5	1
I-0,01	1	6	1
I-0,01	1	7	1
I-0,01	1	8	1
I-0,01	1	9	1
I-0,01	1	10	1
I-0,01	1	11	1
I-0,01	1	12	1
I-0,01	1	13	1
I-0,01	1	14	1
I-0,01	1	15	1
I-0,01	1	16	1
I-0,01	1	17	1
I-0,01	1	18	1
I-0,01	1	19	1
I-0,01	1	20	1
I-0,01	1	21	1
I-0,01	1	22	1
I-0,01	1	23	1
I-0,01	1	24	1
I-0,01	1	25	1

I-0,01	1	26	1
I-0,01	1	27	1
I-0,01	2	1	1
I-0,01	2	2	1
I-0,01	2	3	1
I-0,01	2	4	1
I-0,01	2	5	1
I-0,01	2	6	1
I-0,01	2	7	1
I-0,01	2	8	1
I-0,01	2	9	1
I-0,01	2	10	1
I-0,01	2	11	1
I-0,01	2	12	1
I-0,01	2	13	1
I-0,01	2	14	1
I-0,01	2	15	1
I-0,01	2	16	1
I-0,01	2	17	1
I-0,01	2	18	1
I-0,01	2	19	1
I-0,01	2	20	1
I-0,01	2	21	1
I-0,01	2	22	1
I-0,01	2	23	1
I-0,01	2	24	1
I-0,01	2	25	1
I-0,01	2	26	1
I-0,01	2	27	1
I-0,01	2	28	1
I-0,01	2	29	1
I-0,01	2	30	1
I-0,01	2	31	1
I-0,01	2	32	1
I-0,01	2	33	1
I-0,01	2	34	1
I-0,01	2	35	1
I-0,01	2	36	1
I-0,01	2	37	1
I-0,01	2	38	1
I-0,01	2	39	1
I-0,01	2	40	1
I-0,01	2	41	1
I-0,01	2	42	1
I-0,01	2	43	1

I-0,01	2	44	1
I-0,01	2	45	1
I-0,01	2	46	1
I-0,01	2	47	1
I-0,01	2	48	1
I-0,01	2	49	1
I-0,01	2	50	1
I-0,01	2	51	1
I-0,01	2	52	1
I-0,01	2	53	1
I-0,01	2	54	1
I-0,01	2	55	1
I-0,01	2	56	1
I-0,01	2	57	1
I-0,01	2	58	1
I-0,01	2	59	1
I-0,01	2	60	1
I-0,01	2	61	1
I-0,01	2	62	1
I-0,01	2	63	1
I-0,01	2	64	1
I-0,01	2	65	1
I-0,01	2	66	1
I-0,01	2	67	1
I-0,01	2	68	1
I-0,01	2	69	1
I-0,01	2	70	1
I-0,01	2	71	1
I-0,01	2	72	1
I-0,01	2	73	1
I-0,01	2	74	1
I-0,01	3	1	1
I-0,01	3	2	1
I-0,01	3	3	1
I-0,01	3	4	1
I-0,01	3	5	1
I-0,01	3	6	1
I-0,01	3	7	1
I-0,01	3	8	1
I-0,01	3	9	1
I-0,01	3	10	1
I-0,01	3	11	1
I-0,01	3	12	1
I-0,01	3	13	1
I-0,01	3	14	1

I-0,01	3	15	1
I-0,01	3	16	1
I-0,01	3	17	1
I-0,01	3	18	1
I-0,01	3	19	1
I-0,025	1	1	1
I-0,025	1	2	1
I-0,025	1	3	1
I-0,025	1	4	1
I-0,025	1	5	1
I-0,025	1	6	1
I-0,025	1	7	1
I-0,025	1	8	1
I-0,025	1	9	1
I-0,025	1	10	1
I-0,025	1	11	1
I-0,025	1	12	1
I-0,025	1	13	1
I-0,025	1	14	1
I-0,025	1	15	1
I-0,025	1	16	1
I-0,025	1	17	1
I-0,025	1	18	1
I-0,025	1	19	1
I-0,025	1	20	1
I-0,025	1	21	1
I-0,025	1	22	1
I-0,025	1	23	1
I-0,025	1	24	1
I-0,025	1	25	1
I-0,025	1	26	1
I-0,025	1	27	1
I-0,025	1	28	1
I-0,025	1	29	1
I-0,025	2	1	1
I-0,025	2	2	1
I-0,025	2	3	1
I-0,025	2	4	1
I-0,025	2	5	1
I-0,025	2	6	1
I-0,025	2	7	1
I-0,025	2	8	1
I-0,025	2	9	1
I-0,025	2	10	1
I-0,025	2	11	1

I-0,025	2	12	1
I-0,025	2	13	1
I-0,025	2	14	1
I-0,025	2	15	1
I-0,025	2	16	1
I-0,025	2	17	1
I-0,025	2	18	1
I-0,025	2	19	1
I-0,025	2	20	1
I-0,025	2	21	1
I-0,025	2	22	1
I-0,025	2	23	1
I-0,025	2	24	1
I-0,025	2	25	1
I-0,025	2	26	1
I-0,025	2	27	1
I-0,025	2	28	1
I-0,025	2	29	1
I-0,025	2	30	1
I-0,025	2	31	1
I-0,025	2	32	1
I-0,025	2	33	1
I-0,025	2	34	1
I-0,025	2	35	1
I-0,025	2	36	1
I-0,025	2	37	1
I-0,025	2	38	1
I-0,025	2	39	1
I-0,025	2	40	1
I-0,025	2	41	1
I-0,025	2	42	1
I-0,025	2	43	1
I-0,025	2	44	1
I-0,025	2	45	1
I-0,025	2	46	1
I-0,025	2	47	1
I-0,025	2	48	1
I-0,025	2	49	1
I-0,025	2	50	1
I-0,025	2	51	1
I-0,025	2	52	1
I-0,025	2	53	1
I-0,025	2	54	1
I-0,025	2	55	1
I-0,025	2	56	1

I-0,025	2	57	1
I-0,025	2	58	1
I-0,025	2	59	1
I-0,025	2	60	1
I-0,025	2	61	1
I-0,025	2	62	1
I-0,025	2	63	1
I-0,025	3	1	1
I-0,025	3	2	1
I-0,025	3	3	1
I-0,025	3	4	1
I-0,025	3	5	1
I-0,025	3	6	1
I-0,025	3	7	1
I-0,025	3	8	1
I-0,025	3	9	1
I-0,025	3	10	1
I-0,025	3	11	1
I-0,025	3	12	1
I-0,025	3	13	1
I-0,025	3	14	1
I-0,025	3	15	1
I-0,025	3	16	1
I-0,025	3	17	1
I-0,025	3	18	1
I-0,025	3	19	1
I-0,025	3	20	1
I-0,025	3	21	1
I-0,025	3	22	1
I-0,025	5	1	1
I-0,025	5	2	1
I-0,025	5	3	1
I-0,025	5	4	1
I-0,025	7	1	1
I-0,025	7	2	1
I-0,05	1	1	1
I-0,05	1	2	1
I-0,05	1	3	1
I-0,05	1	4	1
I-0,05	1	5	1
I-0,05	1	6	1
I-0,05	1	7	1
I-0,05	1	8	1
I-0,05	1	9	1
I-0,05	1	10	1

I-0,05	1	11	1
I-0,05	1	12	1
I-0,05	1	13	1
I-0,05	1	14	1
I-0,05	1	15	1
I-0,05	1	16	1
I-0,05	1	17	1
I-0,05	1	18	1
I-0,05	1	19	1
I-0,05	1	20	1
I-0,05	1	21	1
I-0,05	1	22	1
I-0,05	1	23	1
I-0,05	1	24	1
I-0,05	1	25	1
I-0,05	1	26	1
I-0,05	1	27	1
I-0,05	1	28	1
I-0,05	1	29	1
I-0,05	1	30	1
I-0,05	1	31	1
I-0,05	1	32	1
I-0,05	1	33	1
I-0,05	1	34	1
I-0,05	1	35	1
I-0,05	1	36	1
I-0,05	1	37	1
I-0,05	1	38	1
I-0,05	1	39	1
I-0,05	1	40	1
I-0,05	1	41	1
I-0,05	1	42	1
I-0,05	1	43	1
I-0,05	1	44	1
I-0,05	1	45	1
I-0,05	1	46	1
I-0,05	1	47	1
I-0,05	1	48	1
I-0,05	1	49	1
I-0,05	1	50	1
I-0,05	1	51	1
I-0,05	1	52	1
I-0,05	1	53	1
I-0,05	1	54	1
I-0,05	1	55	1

I-0,05	1	56	1
I-0,05	1	57	1
I-0,05	1	58	1
I-0,05	1	59	1
I-0,05	1	60	1
I-0,05	1	61	1
I-0,05	1	62	1
I-0,05	1	63	1
I-0,05	1	64	1
I-0,05	1	65	1
I-0,05	1	66	1
I-0,05	1	67	1
I-0,05	1	68	1
I-0,05	1	69	1
I-0,05	1	70	1
I-0,05	1	71	1
I-0,05	1	72	1
I-0,05	1	73	1
I-0,05	1	74	1
I-0,05	1	75	1
I-0,05	1	76	1
I-0,05	1	77	1
I-0,05	1	78	1
I-0,05	1	79	1
I-0,05	2	1	1
I-0,05	2	2	1
I-0,05	2	3	1
I-0,05	2	4	1
I-0,05	2	5	1
I-0,05	2	6	1
I-0,05	2	7	1
I-0,05	2	8	1
I-0,05	2	9	1
I-0,05	2	10	1
I-0,05	2	11	1
I-0,05	2	12	1
I-0,05	2	13	1
I-0,05	2	14	1
I-0,05	2	15	1
I-0,05	2	16	1
I-0,05	2	17	1
I-0,05	2	18	1
I-0,05	2	19	1
I-0,05	2	20	1
I-0,05	2	21	1

I-0,05	2	22	1
I-0,05	2	23	1
I-0,05	2	24	1
I-0,05	2	25	1
I-0,05	2	26	1
I-0,05	2	27	1
I-0,05	2	28	1
I-0,05	2	29	1
I-0,05	2	30	1
I-0,05	2	31	1
I-0,05	2	32	1
I-0,05	2	33	1
I-0,05	2	34	1
I-0,05	2	35	1
I-0,05	2	36	1
I-0,05	2	37	1
I-0,05	2	38	1
I-0,05	2	39	1
I-0,05	2	40	1
I-0,05	2	41	1
I-0,075	1	1	1
I-0,075	1	2	1
I-0,075	1	3	1
I-0,075	1	4	1
I-0,075	1	5	1
I-0,075	1	6	1
I-0,075	1	7	1
I-0,075	1	8	1
I-0,075	1	9	1
I-0,075	1	10	1
I-0,075	1	11	1
I-0,075	1	12	1
I-0,075	1	13	1
I-0,075	1	14	1
I-0,075	1	15	1
I-0,075	1	16	1
I-0,075	1	17	1
I-0,075	1	18	1
I-0,075	1	19	1
I-0,075	1	20	1
I-0,075	1	21	1
I-0,075	1	22	1
I-0,075	1	23	1
I-0,075	1	24	1
I-0,075	1	25	1

I-0,075	1	26	1
I-0,075	1	27	1
I-0,075	1	28	1
I-0,075	1	29	1
I-0,075	1	30	1
I-0,075	1	31	1
I-0,075	1	32	1
I-0,075	1	33	1
I-0,075	1	34	1
I-0,075	1	35	1
I-0,075	1	36	1
I-0,075	1	37	1
I-0,075	1	38	1
I-0,075	1	39	1
I-0,075	1	40	1
I-0,075	1	41	1
I-0,075	1	42	1
I-0,075	1	43	1
I-0,075	2	1	1
I-0,075	2	2	1
I-0,075	2	3	1
I-0,075	2	4	1
I-0,075	2	5	1
I-0,075	2	6	1
I-0,075	2	7	1
I-0,075	2	8	1
I-0,075	2	9	1
I-0,075	2	10	1
I-0,075	2	11	1
I-0,075	2	12	1
I-0,075	2	13	1
I-0,075	2	14	1
I-0,075	2	15	1
I-0,075	2	16	1
I-0,075	2	17	1
I-0,075	2	18	1
I-0,075	2	19	1
I-0,075	2	20	1
I-0,075	2	21	1
I-0,075	2	22	1
I-0,075	2	23	1
I-0,075	2	24	1
I-0,075	2	25	1
I-0,075	2	26	1
I-0,075	2	27	1

I-0,075	2	28	1
I-0,075	2	29	1
I-0,075	2	30	1
I-0,075	2	31	1
I-0,075	2	32	1
I-0,075	2	33	1
I-0,075	2	34	1
I-0,075	2	35	1
I-0,075	2	36	1
I-0,075	2	37	1
I-0,075	2	38	1
I-0,075	2	39	1
I-0,075	2	40	1
I-0,075	2	41	1
I-0,075	2	42	1
I-0,075	2	43	1
I-0,075	2	44	1
I-0,075	2	45	1
I-0,075	2	46	1
I-0,075	2	47	1
I-0,075	2	48	1
I-0,075	2	49	1
I-0,075	2	50	1
I-0,075	2	51	1
I-0,075	2	52	1
I-0,075	2	53	1
I-0,075	2	54	1
I-0,075	2	55	1
I-0,075	2	56	1
I-0,075	2	57	1
I-0,075	2	58	1
I-0,075	2	59	1
I-0,075	2	60	1
I-0,075	2	61	1
I-0,075	2	62	1
I-0,075	2	63	1
I-0,075	2	64	1
I-0,075	2	65	1
I-0,075	2	66	1
I-0,075	2	67	1
I-0,075	2	68	1
I-0,075	2	69	1
I-0,075	2	70	1
I-0,075	2	71	1
I-0,075	2	72	1

I-0,075	2	73	1
I-0,075	2	74	1
I-0,075	3	1	1
I-0,075	3	2	1
I-0,075	3	3	1
I-0,1	1	1	1
I-0,1	1	2	1
I-0,1	1	3	1
I-0,1	1	4	1
I-0,1	1	5	1
I-0,1	1	6	1
I-0,1	1	7	1
I-0,1	1	8	1
I-0,1	1	9	1
I-0,1	1	10	1
I-0,1	1	11	1
I-0,1	1	12	1
I-0,1	1	13	1
I-0,1	1	14	1
I-0,1	1	15	1
I-0,1	1	16	1
I-0,1	1	17	1
I-0,1	1	18	1
I-0,1	1	19	1
I-0,1	1	20	1
I-0,1	1	21	1
I-0,1	1	22	1
I-0,1	1	23	1
I-0,1	1	24	1
I-0,1	1	25	1
I-0,1	1	26	1
I-0,1	1	27	1
I-0,1	1	28	1
I-0,1	1	29	1
I-0,1	1	30	1
I-0,1	1	31	1
I-0,1	1	32	1
I-0,1	1	33	1
I-0,1	1	34	1
I-0,1	1	35	1
I-0,1	1	36	1
I-0,1	1	37	1
I-0,1	1	38	1
I-0,1	1	39	1
I-0,1	1	40	1

I-0,1	1	41	1
I-0,1	1	42	1
I-0,1	1	43	1
I-0,1	1	44	1
I-0,1	1	45	1
I-0,1	1	46	1
I-0,1	1	47	1
I-0,1	1	48	1
I-0,1	1	49	1
I-0,1	1	50	1
I-0,1	1	51	1
I-0,1	1	52	1
I-0,1	1	53	1
I-0,1	1	54	1
I-0,1	1	55	1
I-0,1	1	56	1
I-0,1	1	57	1
I-0,1	1	58	1
I-0,1	1	59	1
I-0,1	1	60	1
I-0,1	1	61	1
I-0,1	1	62	1
I-0,1	1	63	1
I-0,1	1	64	1
I-0,1	1	65	1
I-0,1	1	66	1
I-0,1	1	67	1
I-0,1	1	68	1
I-0,1	1	69	1
I-0,1	1	70	1
I-0,1	1	71	1
I-0,1	1	72	1
I-0,1	1	73	1
I-0,1	1	74	1
I-0,1	1	75	1
I-0,1	1	76	1
I-0,1	1	77	1
I-0,1	1	78	1
I-0,1	1	79	1
I-0,1	1	80	1
I-0,1	1	81	1
I-0,1	1	82	1
I-0,1	1	83	1
I-0,1	1	84	1
I-0,1	1	85	1

I-0,1	1	86	1
I-0,1	1	87	1
I-0,1	1	88	1
I-0,1	1	89	1
I-0,1	1	90	1
I-0,1	1	91	1
I-0,1	1	92	1
I-0,1	1	93	1
I-0,1	2	1	1
I-0,1	2	2	1
I-0,1	2	3	1
I-0,1	2	4	1
I-0,1	2	5	1
I-0,1	2	6	1
I-0,1	2	7	1
I-0,1	2	8	1
I-0,1	2	9	1
I-0,1	2	10	1
I-0,1	2	11	1
I-0,1	2	12	1
I-0,1	2	13	1
I-0,1	2	14	1
I-0,1	2	15	1
I-0,1	2	16	1
I-0,1	2	17	1
I-0,1	2	18	1
I-0,1	2	19	1
I-0,1	2	20	1
I-0,1	2	21	1
I-0,1	2	22	1
I-0,1	2	23	1
I-0,1	2	24	1
I-0,1	2	25	1
I-0,1	2	26	1
I-0,1	2	27	1
I-0,2	1	1	1
I-0,2	1	2	1
I-0,2	1	3	1
I-0,2	1	4	1
I-0,2	1	5	1
I-0,2	1	6	1
I-0,2	1	7	1
I-0,2	1	8	1
I-0,2	1	9	1
I-0,2	1	10	1

I-0,2	1	11	1
I-0,2	1	12	1
I-0,2	1	13	1
I-0,2	1	14	1
I-0,2	1	15	1
I-0,2	1	16	1
I-0,2	1	17	1
I-0,2	1	18	1
I-0,2	1	19	1
I-0,2	1	20	1
I-0,2	1	21	1
I-0,2	1	22	1
I-0,2	1	23	1
I-0,2	1	24	1
I-0,2	1	25	1
I-0,2	1	26	1
I-0,2	1	27	1
I-0,2	1	28	1
I-0,2	1	29	1
I-0,2	1	30	1
I-0,2	1	31	1
I-0,2	1	32	1
I-0,2	1	33	1
I-0,2	1	34	1
I-0,2	1	35	1
I-0,2	1	36	1
I-0,2	1	37	1
I-0,2	1	38	1
I-0,2	1	39	1
I-0,2	1	40	1
I-0,2	1	41	1
I-0,2	1	42	1
I-0,2	1	43	1
I-0,2	1	44	1
I-0,2	1	45	1
I-0,2	1	46	1
I-0,2	1	47	1
I-0,2	1	48	1
I-0,2	1	49	1
I-0,2	1	50	1
I-0,2	1	51	1
I-0,2	1	52	1
I-0,2	1	53	1
I-0,2	1	54	1
I-0,2	1	55	1

I-0,2	1	56	1
I-0,2	1	57	1
I-0,2	1	58	1
I-0,2	1	59	1
I-0,2	1	60	1
I-0,2	1	61	1
I-0,2	1	62	1
I-0,2	1	63	1
I-0,2	1	64	1
I-0,2	1	65	1
I-0,2	1	66	1
I-0,2	1	67	1
I-0,2	1	68	1
I-0,2	1	69	1
I-0,2	1	70	1
I-0,2	1	71	1
I-0,2	1	72	1
I-0,2	1	73	1
I-0,2	1	74	1
I-0,2	1	75	1
I-0,2	1	76	1
I-0,2	1	77	1
I-0,2	1	78	1
I-0,2	1	79	1
I-0,2	1	80	1
I-0,2	1	81	1
I-0,2	1	82	1
I-0,2	1	83	1
I-0,2	1	84	1
I-0,2	1	85	1
I-0,2	1	86	1
I-0,2	1	87	1
I-0,2	1	88	1
I-0,2	1	89	1
I-0,2	1	90	1
I-0,2	1	91	1
I-0,2	1	92	1
I-0,2	1	93	1
I-0,2	1	94	1
I-0,2	1	95	1
I-0,2	1	96	1
I-0,2	1	97	1
I-0,2	1	98	1
I-0,2	1	99	1
I-0,2	2	1	1

I-0,2	2	2	1
I-0,2	2	3	1
I-0,2	2	4	1
I-0,2	2	5	1
I-0,2	2	6	1
I-0,2	2	7	1
I-0,2	2	8	1
I-0,2	2	9	1
I-0,2	2	10	1
I-0,2	2	11	1
I-0,2	2	12	1
I-0,2	2	13	1
I-0,2	2	14	1
I-0,2	2	15	1
I-0,2	2	16	1
I-0,2	2	17	1
I-0,2	2	18	1
I-0,2	2	19	1
I-0,2	2	20	1
I-0,2	2	21	1
I-0,3	1	1	1
I-0,3	1	2	1
I-0,3	1	3	1
I-0,3	1	4	1
I-0,3	1	5	1
I-0,3	1	6	1
I-0,3	1	7	1
I-0,3	1	8	1
I-0,3	1	9	1
I-0,3	1	10	1
I-0,3	1	11	1
I-0,3	1	12	1
I-0,3	1	13	1
I-0,3	1	14	1
I-0,3	1	15	1
I-0,3	1	16	1
I-0,3	1	17	1
I-0,3	1	18	1
I-0,3	1	19	1
I-0,3	1	20	1
I-0,3	1	21	1
I-0,3	1	22	1
I-0,3	1	23	1
I-0,3	1	24	1
I-0,3	1	25	1

I-0,3	1	26	1
I-0,3	1	27	1
I-0,3	1	28	1
I-0,3	1	29	1
I-0,3	1	30	1
I-0,3	1	31	1
I-0,3	1	32	1
I-0,3	1	33	1
I-0,3	1	34	1
I-0,3	1	35	1
I-0,3	1	36	1
I-0,3	1	37	1
I-0,3	1	38	1
I-0,3	1	39	1
I-0,3	1	40	1
I-0,3	1	41	1
I-0,3	1	42	1
I-0,3	1	43	1
I-0,3	1	44	1
I-0,3	1	45	1
I-0,3	1	46	1
I-0,3	1	47	1
I-0,3	1	48	1
I-0,3	1	49	1
I-0,3	1	50	1
I-0,3	1	51	1
I-0,3	1	52	1
I-0,3	1	53	1
I-0,3	1	54	1
I-0,3	1	55	1
I-0,3	1	56	1
I-0,3	1	57	1
I-0,3	1	58	1
I-0,3	1	59	1
I-0,3	1	60	1
I-0,3	1	61	1
I-0,3	1	62	1
I-0,3	1	63	1
I-0,3	1	64	1
I-0,3	1	65	1
I-0,3	1	66	1
I-0,3	1	67	1
I-0,3	1	68	1
I-0,3	1	69	1
I-0,3	1	70	1

I-0,3	1	71	1
I-0,3	1	72	1
I-0,3	1	73	1
I-0,3	1	74	1
I-0,3	1	75	1
I-0,3	1	76	1
I-0,3	1	77	1
I-0,3	1	78	1
I-0,3	1	79	1
I-0,3	1	80	1
I-0,3	1	81	1
I-0,3	1	82	1
I-0,3	1	83	1
I-0,3	1	84	1
I-0,3	1	85	1
I-0,3	1	86	1
I-0,3	1	87	1
I-0,3	1	88	1
I-0,3	1	89	1
I-0,3	1	90	1
I-0,3	1	91	1
I-0,3	1	92	1
I-0,3	1	93	1
I-0,3	1	94	1
I-0,3	1	95	1
I-0,3	1	96	1
I-0,3	1	97	1
I-0,3	1	98	1
I-0,3	1	99	1
I-0,3	1	100	1
I-0,3	1	101	1
I-0,3	1	102	1
I-0,3	1	103	1
I-0,3	1	104	1
I-0,3	1	105	1
I-0,3	1	106	1
I-0,3	1	107	1
I-0,3	1	108	1
I-0,3	1	109	1
I-0,3	1	110	1
I-0,3	1	111	1
I-0,3	1	112	1
I-0,3	1	113	1
I-0,3	1	114	1
I-0,3	1	115	1

I-0,3	1	116	1
I-0,3	1	117	1
I-0,3	1	118	1
I-0,3	2	1	1
I-0,3	2	2	1
placebo	3	1	1
placebo	5	1	1
placebo	5	2	1
placebo	5	3	1
placebo	5	4	1
placebo	5	5	1
placebo	5	6	1
placebo	5	7	1
placebo	5	8	1
placebo	5	9	1
placebo	5	10	1
placebo	5	11	1
placebo	5	12	1
placebo	5	13	1
placebo	7	1	1
placebo	7	2	1
placebo	7	3	1
placebo	7	4	1
placebo	7	5	1
placebo	7	6	1
placebo	7	7	1
placebo	7	8	1
placebo	7	9	1
placebo	7	10	1
placebo	7	11	1
placebo	7	12	1
placebo	7	13	1
placebo	7	14	1
placebo	21	1	0
placebo	21	2	0
placebo	21	3	0
placebo	21	4	0
placebo	21	5	0
placebo	21	6	0
placebo	21	7	0
placebo	21	8	0
placebo	21	9	0
placebo	21	10	0
placebo	21	11	0
placebo	21	12	0

placebo	21	13	0
placebo	21	14	0
placebo	21	15	0
placebo	21	16	0
placebo	21	17	0
placebo	21	18	0
placebo	21	19	0
placebo	21	20	0
placebo	21	21	0
placebo	21	22	0
placebo	21	23	0
placebo	21	24	0
placebo	21	25	0
placebo	21	26	0
placebo	21	27	0
placebo	21	28	0
placebo	21	29	0
placebo	21	30	0
placebo	21	31	0
placebo	21	32	0
placebo	21	33	0
placebo	21	34	0
placebo	21	35	0
placebo	21	36	0
placebo	21	37	0
placebo	21	38	0
placebo	21	39	0
placebo	21	40	0
placebo	21	41	0
placebo	21	42	0
placebo	21	43	0
placebo	21	44	0
placebo	21	45	0
placebo	21	46	0
placebo	21	47	0
placebo	21	48	0
placebo	21	49	0
placebo	21	50	0
placebo	21	51	0
placebo	21	52	0
placebo	21	53	0
placebo	21	54	0
placebo	21	55	0
placebo	21	56	0
placebo	21	57	0

placebo	21	58	0
placebo	21	59	0
placebo	21	60	0
placebo	21	61	0
placebo	21	62	0
placebo	21	63	0
placebo	21	64	0
placebo	21	65	0
placebo	21	66	0
placebo	21	67	0
placebo	21	68	0
placebo	21	69	0
placebo	21	70	0
placebo	21	71	0
placebo	21	72	0
placebo	21	73	0
placebo	21	74	0
placebo	21	75	0
placebo	21	76	0
placebo	21	77	0
placebo	21	78	0
placebo	21	79	0
placebo	21	80	0
placebo	21	81	0
placebo	21	82	0
placebo	21	83	0
placebo	21	84	0
placebo	21	85	0
placebo	21	86	0
placebo	21	87	0
placebo	21	88	0
placebo	21	89	0
placebo	21	90	0
placebo	21	91	0
placebo	21	92	0

Bioassay with laboratory colonies - Fig3

treatment	repetition	day	carried%	incorporated%	returned%
I-0,001	1	1	100	100	0
I-0,001	1	2	100	100	0
I-0,001	2	1	100	100	0
I-0,001	2	2	100	100	0
I-0,001	3	1	75	100	0
I-0,001	3	2	100	100	0
I-0,001	4	1	100	100	0
I-0,001	4	2	100	100	0
I-0,001	5	1	100	100	0
I-0,001	5	2	100	100	0
I-0,003	1	1	100	100	0
I-0,003	1	2	100	100	0
I-0,003	2	1	100	100	0
I-0,003	2	2	100	100	0
I-0,003	3	1	100	100	0
I-0,003	3	2	100	100	0
I-0,003	4	1	100	100	0
I-0,003	4	2	100	100	0
I-0,003	5	1	100	100	0
I-0,003	5	2	100	100	0
I-0,025	1	1	25	25	0
I-0,025	1	2	25	25	0
I-0,025	2	1	100	100	0
I-0,025	2	2	100	100	0
I-0,025	3	1	100	100	0
I-0,025	3	2	100	100	0
I-0,025	4	1	100	100	0
I-0,025	4	2	100	100	0
I-0,025	5	1	100	100	0
I-0,025	5	2	100	100	0
I-0,03	1	1	25	75	0
I-0,03	1	2	25	100	0
I-0,03	2	1	100	100	0
I-0,03	2	2	100	100	0
I-0,03	3	1	100	100	0
I-0,03	3	2	100	100	0
I-0,03	4	1	100	100	0
I-0,03	4	2	100	100	0
I-0,03	5	1	100	100	0
I-0,03	5	2	100	100	0
I-0,05	1	1	100	100	0
I-0,05	1	2	100	100	0

I-0,05	2	1	25	100	0
I-0,05	2	2	25	100	0
I-0,05	3	1	100	50	0
I-0,05	3	2	100	50	0
I-0,05	4	1	100	50	0
I-0,05	4	2	100	50	0
I-0,05	5	1	50	50	0
I-0,05	5	2	50	100	0
I-0,075	1	1	100	75	0
I-0,075	1	2	100	100	0
I-0,075	2	1	25	100	0
I-0,075	2	2	25	100	0
I-0,075	3	1	100	50	0
I-0,075	3	2	100	100	0
I-0,075	4	1	100	50	0
I-0,075	4	2	100	50	0
I-0,075	5	1	25	25	0
I-0,075	5	2	25	100	0
I-0,1	1	1	75	50	0
I-0,1	1	2	100	100	0
I-0,1	2	1	25	100	0
I-0,1	2	2	25	100	0
I-0,1	3	1	25	50	0
I-0,1	3	2	25	50	0
I-0,1	4	1	25	100	0
I-0,1	4	2	25	100	0
I-0,1	5	1	25	100	0
I-0,1	5	2	25	100	0
I-0,2	1	1	50	50	0
I-0,2	1	2	50	50	0
I-0,2	2	1	25	70	0
I-0,2	2	2	25	70	0
I-0,2	3	1	25	70	0
I-0,2	3	2	25	70	0
I-0,2	4	1	25	90	0
I-0,2	4	2	25	90	0
I-0,2	5	1	25	70	0
I-0,2	5	2	25	70	0
I-0,3	1	1	100	100	0
I-0,3	1	2	100	100	0
I-0,3	2	1	25	100	0
I-0,3	2	2	25	100	0
I-0,3	3	1	25	100	0
I-0,3	3	2	25	100	0
I-0,3	4	1	25	100	0

I-0,3	4	2	25	100	0
I-0,3	5	1	25	100	0
I-0,3	5	2	25	100	0
control	1	1	75	100	0
control	1	2	75	100	0
control	2	1	75	100	0
control	2	2	75	100	0
control	3	1	100	100	0
control	3	2	100	100	0
control	4	1	75	100	0
control	4	2	100	100	0
control	5	1	25	100	0
control	5	2	100	100	0
placebo	1	1	100	100	0
placebo	1	2	100	100	0
placebo	2	1	100	100	0
placebo	2	2	100	100	0
placebo	3	1	100	100	0
placebo	3	2	100	100	0
placebo	4	1	25	25	0
placebo	4	2	25	25	0
placebo	5	1	25	100	0
placebo	5	2	100	100	0

Bioassay with laboratory colonies - Fig4 and Fig5

treatment	repetition	day	cutting%	cutting	mortality
I-0,001	1	1	100	1	0
I-0,001	2	1	100	1	0
I-0,001	3	1	100	1	0
I-0,001	4	1	100	1	0
I-0,001	5	1	100	1	0
I-0,001	1	2	100	1	0
I-0,001	2	2	100	1	0
I-0,001	3	2	100	1	0
I-0,001	4	2	100	1	0
I-0,001	5	2	100	1	0
I-0,001	1	3	25	1	0
I-0,001	2	3	25	1	0
I-0,001	3	3	0	0	0
I-0,001	4	3	0	0	0
I-0,001	5	3	0	0	0
I-0,001	1	6	50	1	0
I-0,001	2	6	0	0	0
I-0,001	3	6	0	0	0
I-0,001	4	6	0	0	0
I-0,001	5	6	0	0	0
I-0,001	1	9	50	1	0
I-0,001	2	9	50	1	0
I-0,001	3	9	25	1	0
I-0,001	4	9	0	0	0
I-0,001	5	9	0	0	0
I-0,001	1	12	50	1	0
I-0,001	2	12	75	1	0
I-0,001	3	12	0	0	0
I-0,001	4	12	0	0	0
I-0,001	5	12	0	0	0
I-0,001	1	15	100	1	0
I-0,001	2	15	100	1	0
I-0,001	3	15	75	1	0
I-0,001	4	15	0	0	0
I-0,001	5	15	0	0	0
I-0,001	1	18	100	1	0
I-0,001	2	18	100	1	0
I-0,001	3	18	25	1	0
I-0,001	4	18	100	1	0
I-0,001	5	18	100	1	0
I-0,001	1	21	100	1	0
I-0,001	2	21	100	1	0

I-0,001	3	21	50	1	0
I-0,001	4	21	100	1	0
I-0,001	5	21	100	1	0
I-0,001	1	24	100	1	0
I-0,001	2	24	100	1	0
I-0,001	3	24	100	1	0
I-0,001	4	24	100	1	0
I-0,001	5	24	100	1	0
I-0,001	1	27	100	1	0
I-0,001	2	27	100	1	0
I-0,001	3	27	100	1	0
I-0,001	4	27	75	1	0
I-0,001	5	27	100	1	0
I-0,001	1	30	75	1	0
I-0,001	2	30	100	1	0
I-0,001	3	30	75	1	0
I-0,001	4	30	100	1	0
I-0,001	5	30	0	0	0
I-0,001	1	33	75	1	0
I-0,001	2	33	100	1	0
I-0,001	3	33	100	1	0
I-0,001	4	33	100	1	0
I-0,001	5	33	50	1	0
I-0,001	1	36	100	1	0
I-0,001	2	36	100	1	0
I-0,001	3	36	75	1	0
I-0,001	4	36	100	1	0
I-0,001	5	36	75	1	0
I-0,001	1	42	100	1	0
I-0,001	2	42	100	1	0
I-0,001	3	42	75	1	0
I-0,001	4	42	100	1	0
I-0,001	5	42	75	1	0
I-0,003	1	1	100	1	0
I-0,003	2	1	100	1	0
I-0,003	3	1	100	1	0
I-0,003	4	1	100	1	0
I-0,003	5	1	100	1	0
I-0,003	1	2	100	1	0
I-0,003	2	2	100	1	0
I-0,003	3	2	100	1	0
I-0,003	4	2	100	1	0
I-0,003	5	2	100	1	0
I-0,003	1	3	50	1	0
I-0,003	2	3	75	1	0

I-0,003	3	3	100	1	0
I-0,003	4	3	100	1	0
I-0,003	5	3	75	1	0
I-0,003	1	6	0	0	0
I-0,003	2	6	75	1	0
I-0,003	3	6	100	1	0
I-0,003	4	6	50	1	0
I-0,003	5	6	25	1	0
I-0,003	1	9	75	1	0
I-0,003	2	9	100	1	0
I-0,003	3	9	100	1	0
I-0,003	4	9	100	1	0
I-0,003	5	9	100	1	0
I-0,003	1	12	100	1	0
I-0,003	2	12	100	1	0
I-0,003	3	12	100	1	0
I-0,003	4	12	100	1	0
I-0,003	5	12	100	1	0
I-0,003	1	15	100	1	0
I-0,003	2	15	100	1	0
I-0,003	3	15	100	1	0
I-0,003	4	15	100	1	0
I-0,003	5	15	100	1	0
I-0,003	1	18	75	1	0
I-0,003	2	18	100	1	0
I-0,003	3	18	100	1	0
I-0,003	4	18	100	1	0
I-0,003	5	18	100	1	0
I-0,003	1	21	100	1	0
I-0,003	2	21	100	1	0
I-0,003	3	21	100	1	0
I-0,003	4	21	100	1	0
I-0,003	5	21	100	1	0
I-0,003	1	24	100	1	0
I-0,003	2	24	100	1	0
I-0,003	3	24	100	1	0
I-0,003	4	24	100	1	0
I-0,003	5	24	100	1	0
I-0,003	1	27	75	1	0
I-0,003	2	27	50	1	0
I-0,003	3	27	100	1	0
I-0,003	4	27	100	1	0
I-0,003	5	27	100	1	0
I-0,003	1	30	100	1	0
I-0,003	2	30	75	1	0

I-0,003	3	30	100	1	0
I-0,003	4	30	100	1	0
I-0,003	5	30	50	1	0
I-0,003	1	33	100	1	0
I-0,003	2	33	25	1	0
I-0,003	3	33	50	1	0
I-0,003	4	33	75	1	0
I-0,003	5	33	75	1	0
I-0,003	1	36	100	1	0
I-0,003	2	36	100	1	0
I-0,003	3	36	100	1	0
I-0,003	4	36	100	1	0
I-0,003	5	36	100	1	0
I-0,003	1	42	100	1	0
I-0,003	2	42	100	1	0
I-0,003	3	42	100	1	0
I-0,003	4	42	100	1	0
I-0,003	5	42	100	1	0
I-0,025	1	1	100	1	0
I-0,025	2	1	100	1	0
I-0,025	3	1	100	1	0
I-0,025	4	1	100	1	0
I-0,025	5	1	100	1	0
I-0,025	1	2	100	1	0
I-0,025	2	2	100	1	0
I-0,025	3	2	100	1	0
I-0,025	4	2	100	1	0
I-0,025	5	2	100	1	0
I-0,025	1	3	0	0	0
I-0,025	2	3	0	0	0
I-0,025	3	3	0	0	0
I-0,025	4	3	0	0	0
I-0,025	5	3	0	0	0
I-0,025	1	6	25	1	0
I-0,025	2	6	0	0	0
I-0,025	3	6	0	0	0
I-0,025	4	6	0	0	0
I-0,025	5	6	0	0	0
I-0,025	1	9	25	1	0
I-0,025	2	9	0	0	0
I-0,025	3	9	25	1	0
I-0,025	4	9	0	0	0
I-0,025	5	9	25	1	0
I-0,025	1	12	25	1	0
I-0,025	2	12	0	0	0

-0,025	3	12	0	0	0
I-0,025	4	12	0	0	0
I-0,025	5	12	25	1	0
I-0,025	1	15	50	1	0
I-0,025	2	15	0	0	0
I-0,025	3	15	0	0	0
I-0,025	4	15	25	1	0
I-0,025	5	15	0	0	0
I-0,025	1	18	50	1	0
I-0,025	2	18	0	0	0
I-0,025	3	18	0	0	0
I-0,025	4	18	0	0	0
I-0,025	5	18	0	0	0
I-0,025	1	21	25	1	0
I-0,025	2	21	0	0	0
I-0,025	3	21	0	0	0
I-0,025	4	21	0	0	0
I-0,025	5	21	0	0	0
I-0,025	1	24	25	1	0
I-0,025	2	24	0	0	0
I-0,025	3	24	0	0	0
I-0,025	4	24	0	0	0
I-0,025	5	24	0	0	0
I-0,025	1	27	25	1	0
I-0,025	2	27	0	0	0
I-0,025	3	27	0	0	0
I-0,025	4	27	0	0	0
I-0,025	5	27	0	0	0
I-0,025	1	30	50	1	0
I-0,025	2	30	0	0	0
I-0,025	3	30	0	0	0
I-0,025	4	30	0	0	0
I-0,025	5	30	0	0	0
I-0,025	1	33	25	1	0
I-0,025	2	33	0	0	0
I-0,025	3	33	0	0	0
I-0,025	4	33	0	0	0
I-0,025	5	33	0	0	0
I-0,025	1	36	0	0	0
I-0,025	2	36	0	0	0
I-0,025	3	36	0	0	0
I-0,025	4	36	0	0	0
I-0,025	5	36	0	0	0
I-0,025	1	42	0	0	0
I-0,025	2	42	0	0	0

I-0,025	3	42	0	0	0
I-0,025	4	42	0	0	0
I-0,025	5	42	0	0	0
I-0,03	1	1	100	1	0
I-0,03	2	1	100	1	0
I-0,03	3	1	100	1	0
I-0,03	4	1	100	1	0
I-0,03	5	1	100	1	0
I-0,03	1	2	100	1	0
I-0,03	2	2	100	1	0
I-0,03	3	2	100	1	0
I-0,03	4	2	100	1	0
I-0,03	5	2	100	1	0
I-0,03	1	3	25	1	0
I-0,03	2	3	50	1	0
I-0,03	3	3	75	1	0
I-0,03	4	3	100	1	0
I-0,03	5	3	100	1	0
I-0,03	1	6	25	1	0
I-0,03	2	6	0	0	0
I-0,03	3	6	25	1	0
I-0,03	4	6	100	1	0
I-0,03	5	6	100	1	0
I-0,03	1	9	100	1	0
I-0,03	2	9	25	1	0
I-0,03	3	9	100	1	0
I-0,03	4	9	100	1	0
I-0,03	5	9	100	1	0
I-0,03	1	12	100	1	0
I-0,03	2	12	50	1	0
I-0,03	3	12	75	1	0
I-0,03	4	12	100	1	0
I-0,03	5	12	75	1	0
I-0,03	1	15	100	1	0
I-0,03	2	15	50	1	0
I-0,03	3	15	100	1	0
I-0,03	4	15	100	1	0
I-0,03	5	15	100	1	0
I-0,03	1	18	100	1	0
I-0,03	2	18	100	1	1
I-0,03	3	18	100	1	1
I-0,03	4	18	100	1	0
I-0,03	5	18	100	1	1
I-0,03	1	21	100	1	0
I-0,03	2	21	100	1	1

I-0,03	3	21	100	1	1
I-0,03	4	21	100	1	0
I-0,03	5	21	100	1	1
I-0,03	1	24	100	1	0
I-0,03	2	24	100	1	1
I-0,03	3	24	100	1	1
I-0,03	4	24	100	1	0
I-0,03	5	24	100	1	1
I-0,03	1	27	100	1	0
I-0,03	2	27	100	1	1
I-0,03	3	27	100	1	1
I-0,03	4	27	100	1	0
I-0,03	5	27	100	1	1
I-0,03	1	30	100	1	0
I-0,03	2	30	75	1	1
I-0,03	3	30	50	1	1
I-0,03	4	30	100	1	0
I-0,03	5	30	100	1	1
I-0,03	1	33	75	1	0
I-0,03	2	33	25	1	1
I-0,03	3	33	75	1	1
I-0,03	4	33	100	1	0
I-0,03	5	33	100	1	1
I-0,03	1	36	25	1	0
I-0,03	2	36	50	1	1
I-0,03	3	36	100	1	1
I-0,03	4	36	100	1	1
I-0,03	5	36	75	1	1
I-0,03	1	42	25	1	0
I-0,03	2	42	50	1	1
I-0,03	3	42	100	1	1
I-0,03	4	42	100	1	1
I-0,03	5	42	75	1	1
I-0,05	1	1	100	1	0
I-0,05	2	1	100	1	0
I-0,05	3	1	100	1	0
I-0,05	4	1	100	1	0
I-0,05	5	1	100	1	0
I-0,05	1	2	100	1	0
I-0,05	2	2	100	1	0
I-0,05	3	2	100	1	0
I-0,05	4	2	100	1	0
I-0,05	5	2	100	1	0
I-0,05	1	3	0	0	0
I-0,05	2	3	0	0	0

I-0,05	3	3	25	1	0
I-0,05	4	3	25	1	0
I-0,05	5	3	0	0	0
I-0,05	1	6	0	0	0
I-0,05	2	6	25	1	0
I-0,05	3	6	25	1	0
I-0,05	4	6	0	0	0
I-0,05	5	6	0	0	0
I-0,05	1	9	0	0	0
I-0,05	2	9	0	0	0
I-0,05	3	9	0	0	0
I-0,05	4	9	0	0	0
I-0,05	5	9	0	0	0
I-0,05	1	12	0	0	1
I-0,05	2	12	0	0	0
I-0,05	3	12	0	0	0
I-0,05	4	12	0	0	0
I-0,05	5	12	0	0	0
I-0,05	1	15	0	0	1
I-0,05	2	15	0	0	0
I-0,05	3	15	0	0	0
I-0,05	4	15	0	0	0
I-0,05	5	15	0	0	0
I-0,05	1	18	0	0	1
I-0,05	2	18	25	1	0
I-0,05	3	18	0	0	1
I-0,05	4	18	0	0	1
I-0,05	5	18	0	0	1
I-0,05	1	21	0	0	1
I-0,05	2	21	75	1	0
I-0,05	3	21	0	0	1
I-0,05	4	21	0	0	1
I-0,05	5	21	0	0	1
I-0,05	1	24	0	0	1
I-0,05	2	24	25	1	0
I-0,05	3	24	0	0	1
I-0,05	4	24	0	0	1
I-0,05	5	24	0	0	1
I-0,05	1	27	0	0	1
I-0,05	2	27	25	1	0
I-0,05	3	27	0	0	1
I-0,05	4	27	0	0	1
I-0,05	5	27	0	0	1
I-0,05	1	30	0	0	1
I-0,05	2	30	25	1	0

I-0,05	3	30	0	0	1
I-0,05	4	30	0	0	1
I-0,05	5	30	0	0	1
I-0,05	1	33	0	0	1
I-0,05	2	33	0	0	0
I-0,05	3	33	0	0	1
I-0,05	4	33	0	0	1
I-0,05	5	33	0	0	1
I-0,05	1	36	0	0	1
I-0,05	2	36	25	1	0
I-0,05	3	36	0	0	1
I-0,05	4	36	0	0	1
I-0,05	5	36	0	0	1
I-0,05	1	42	0	0	1
I-0,05	2	42	25	1	0
I-0,05	3	42	0	0	1
I-0,05	4	42	0	0	1
I-0,05	5	42	0	0	1
I-0,075	1	1	100	1	0
I-0,075	2	1	100	1	0
I-0,075	3	1	100	1	0
I-0,075	4	1	100	1	0
I-0,075	5	1	100	1	0
I-0,075	1	2	100	1	0
I-0,075	2	2	100	1	0
I-0,075	3	2	100	1	0
I-0,075	4	2	100	1	0
I-0,075	5	2	100	1	0
I-0,075	1	3	0	0	0
I-0,075	2	3	0	0	0
I-0,075	3	3	0	0	0
I-0,075	4	3	25	1	0
I-0,075	5	3	0	0	0
I-0,075	1	6	0	0	0
I-0,075	2	6	0	0	0
I-0,075	3	6	0	0	0
I-0,075	4	6	0	0	0
I-0,075	5	6	0	0	0
I-0,075	1	9	25	1	0
I-0,075	2	9	0	0	0
I-0,075	3	9	0	0	0
I-0,075	4	9	25	1	0
I-0,075	5	9	0	0	0
I-0,075	1	12	0	0	0
I-0,075	2	12	0	0	0

I-0,075	3	12	0	0	0
I-0,075	4	12	0	0	0
I-0,075	5	12	0	0	0
I-0,075	1	15	0	0	0
I-0,075	2	15	0	0	0
I-0,075	3	15	0	0	0
I-0,075	4	15	0	0	0
I-0,075	5	15	0	0	0
I-0,075	1	18	0	0	1
I-0,075	2	18	25	1	0
I-0,075	3	18	0	0	1
I-0,075	4	18	0	0	1
I-0,075	5	18	0	0	1
I-0,075	1	21	0	0	1
I-0,075	2	21	25	1	0
I-0,075	3	21	0	0	1
I-0,075	4	21	0	0	1
I-0,075	5	21	0	0	1
I-0,075	1	24	0	0	1
I-0,075	2	24	0	0	0
I-0,075	3	24	0	0	1
I-0,075	4	24	0	0	1
I-0,075	5	24	0	0	1
I-0,075	1	27	0	0	1
I-0,075	2	27	0	0	0
I-0,075	3	27	0	0	1
I-0,075	4	27	0	0	1
I-0,075	5	27	0	0	1
I-0,075	1	30	0	0	1
I-0,075	2	30	50	1	0
I-0,075	3	30	0	0	1
I-0,075	4	30	0	0	1
I-0,075	5	30	0	0	1
I-0,075	1	33	0	0	1
I-0,075	2	33	25	1	0
I-0,075	3	33	0	0	1
I-0,075	4	33	0	0	1
I-0,075	5	33	0	0	1
I-0,075	1	36	0	0	1
I-0,075	2	36	0	0	0
I-0,075	3	36	0	0	1
I-0,075	4	36	0	0	1
I-0,075	5	36	0	0	1
I-0,075	1	42	0	0	1
I-0,075	2	42	0	0	0

I-0,075	3	42	0	0	1
I-0,075	4	42	0	0	1
I-0,075	5	42	0	0	1
I-0,1	1	1	100	1	0
I-0,1	2	1	100	1	0
I-0,1	3	1	100	1	0
I-0,1	4	1	100	1	0
I-0,1	5	1	100	1	0
I-0,1	1	2	100	1	0
I-0,1	2	2	100	1	0
I-0,1	3	2	100	1	0
I-0,1	4	2	100	1	0
I-0,1	5	2	100	1	0
I-0,1	1	3	0	0	0
I-0,1	2	3	0	0	0
I-0,1	3	3	0	0	0
I-0,1	4	3	0	0	0
I-0,1	5	3	0	0	0
I-0,1	1	6	25	1	0
I-0,1	2	6	0	0	0
I-0,1	3	6	0	0	0
I-0,1	4	6	0	0	0
I-0,1	5	6	0	0	0
I-0,1	1	9	0	0	0
I-0,1	2	9	0	0	0
I-0,1	3	9	0	0	0
I-0,1	4	9	0	0	0
I-0,1	5	9	0	0	0
I-0,1	1	12	100	1	0
I-0,1	2	12	0	0	0
I-0,1	3	12	0	0	0
I-0,1	4	12	0	0	0
I-0,1	5	12	0	0	0
I-0,1	1	15	100	1	0
I-0,1	2	15	0	0	0
I-0,1	3	15	0	0	0
I-0,1	4	15	0	0	0
I-0,1	5	15	0	0	0
I-0,1	1	18	0	0	1
I-0,1	2	18	0	0	0
I-0,1	3	18	0	0	1
I-0,1	4	18	0	0	1
I-0,1	5	18	0	0	0
I-0,1	1	21	0	0	1
I-0,1	2	21	0	0	0

I-0,1	3	21	0	0	1
I-0,1	4	21	0	0	1
I-0,1	5	21	0	0	0
I-0,1	1	24	0	0	1
I-0,1	2	24	25	1	0
I-0,1	3	24	0	0	1
I-0,1	4	24	0	0	1
I-0,1	5	24	25	1	0
I-0,1	1	27	0	0	1
I-0,1	2	27	50	1	0
I-0,1	3	27	0	0	1
I-0,1	4	27	0	0	1
I-0,1	5	27	50	1	0
I-0,1	1	30	0	0	1
I-0,1	2	30	0	0	0
I-0,1	3	30	0	0	1
I-0,1	4	30	0	0	1
I-0,1	5	30	25	1	0
I-0,1	1	33	0	0	1
I-0,1	2	33	0	0	1
I-0,1	3	33	0	0	1
I-0,1	4	33	0	0	1
I-0,1	5	33	0	0	0
I-0,1	1	36	0	0	1
I-0,1	2	36	0	0	1
I-0,1	3	36	0	0	1
I-0,1	4	36	0	0	1
I-0,1	5	36	0	0	0
I-0,1	1	42	0	0	1
I-0,1	2	42	0	0	1
I-0,1	3	42	0	0	1
I-0,1	4	42	0	0	1
I-0,1	5	42	0	0	0
I-0,2	1	1	100	1	0
I-0,2	2	1	100	1	0
I-0,2	3	1	100	1	0
I-0,2	4	1	100	1	0
I-0,2	5	1	100	1	0
I-0,2	1	2	100	1	0
I-0,2	2	2	100	1	0
I-0,2	3	2	100	1	0
I-0,2	4	2	100	1	0
I-0,2	5	2	100	1	0
I-0,2	1	3	50	1	0
I-0,2	2	3	0	0	0

I-0,2	3	3	0	0	0
I-0,2	4	3	0	0	0
I-0,2	5	3	0	0	0
I-0,2	1	6	25	1	0
I-0,2	2	6	0	0	0
I-0,2	3	6	0	0	0
I-0,2	4	6	0	0	0
I-0,2	5	6	0	0	0
I-0,2	1	9	50	1	0
I-0,2	2	9	25	1	0
I-0,2	3	9	0	0	0
I-0,2	4	9	50	1	0
I-0,2	5	9	0	0	0
I-0,2	1	12	100	1	0
I-0,2	2	12	25	1	0
I-0,2	3	12	0	0	0
I-0,2	4	12	0	0	0
I-0,2	5	12	0	0	0
I-0,2	1	15	0	0	0
I-0,2	2	15	0	0	0
I-0,2	3	15	0	0	0
I-0,2	4	15	0	0	0
I-0,2	5	15	0	0	0
I-0,2	1	18	0	0	1
I-0,2	2	18	25	1	0
I-0,2	3	18	0	0	1
I-0,2	4	18	0	0	1
I-0,2	5	18	25	1	0
I-0,2	1	21	0	0	1
I-0,2	2	21	50	1	0
I-0,2	3	21	25	1	1
I-0,2	4	21	0	0	1
I-0,2	5	21	50	1	0
I-0,2	1	24	0	0	1
I-0,2	2	24	50	1	0
I-0,2	3	24	0	0	1
I-0,2	4	24	25	1	1
I-0,2	5	24	25	1	0
I-0,2	1	27	0	0	1
I-0,2	2	27	0	0	0
I-0,2	3	27	0	0	1
I-0,2	4	27	0	0	1
I-0,2	5	27	0	0	0
I-0,2	1	30	0	0	1
I-0,2	2	30	0	0	0

I-0,2	3	30	0	0	1
I-0,2	4	30	0	0	1
I-0,2	5	30	0	0	0
I-0,2	1	33	0	0	1
I-0,2	2	33	25	1	1
I-0,2	3	33	0	0	1
I-0,2	4	33	0	0	1
I-0,2	5	33	25	1	1
I-0,2	1	36	0	0	1
I-0,2	2	36	0	0	1
I-0,2	3	36	0	0	1
I-0,2	4	36	0	0	1
I-0,2	5	36	0	0	1
I-0,2	1	42	0	0	1
I-0,2	2	42	0	0	1
I-0,2	3	42	0	0	1
I-0,2	4	42	0	0	1
I-0,2	5	42	0	0	1
I-0,3	1	1	100	1	0
I-0,3	2	1	100	1	0
I-0,3	3	1	100	1	0
I-0,3	4	1	100	1	0
I-0,3	5	1	100	1	0
I-0,3	1	2	100	1	0
I-0,3	2	2	100	1	0
I-0,3	3	2	100	1	0
I-0,3	4	2	100	1	0
I-0,3	5	2	100	1	0
I-0,3	1	3	0	0	0
I-0,3	2	3	25	1	0
I-0,3	3	3	25	1	0
I-0,3	4	3	25	1	0
I-0,3	5	3	0	0	0
I-0,3	1	6	0	0	0
I-0,3	2	6	0	0	0
I-0,3	3	6	0	0	0
I-0,3	4	6	0	0	0
I-0,3	5	6	0	0	0
I-0,3	1	9	0	0	0
I-0,3	2	9	0	0	0
I-0,3	3	9	0	0	0
I-0,3	4	9	0	0	0
I-0,3	5	9	0	0	0
I-0,3	1	12	0	0	1
I-0,3	2	12	0	0	1

I-0,3	3	12	0	0	1
I-0,3	4	12	0	0	1
I-0,3	5	12	0	0	1
I-0,3	1	15	0	0	1
I-0,3	2	15	0	0	1
I-0,3	3	15	0	0	1
I-0,3	4	15	0	0	1
I-0,3	5	15	0	0	1
I-0,3	1	18	0	0	1
I-0,3	2	18	0	0	1
I-0,3	3	18	0	0	1
I-0,3	4	18	0	0	1
I-0,3	5	18	0	0	1
I-0,3	1	21	0	0	1
I-0,3	2	21	0	0	1
I-0,3	3	21	0	0	1
I-0,3	4	21	0	0	1
I-0,3	5	21	0	0	1
I-0,3	1	24	0	0	1
I-0,3	2	24	0	0	1
I-0,3	3	24	0	0	1
I-0,3	4	24	0	0	1
I-0,3	5	24	0	0	1
I-0,3	1	27	0	0	1
I-0,3	2	27	0	0	1
I-0,3	3	27	0	0	1
I-0,3	4	27	0	0	1
I-0,3	5	27	0	0	1
I-0,3	1	30	0	0	1
I-0,3	2	30	0	0	1
I-0,3	3	30	0	0	1
I-0,3	4	30	0	0	1
I-0,3	5	30	0	0	1
I-0,3	1	33	0	0	1
I-0,3	2	33	0	0	1
I-0,3	3	33	0	0	1
I-0,3	4	33	0	0	1
I-0,3	5	33	0	0	1
I-0,3	1	36	0	0	1
I-0,3	2	36	0	0	1
I-0,3	3	36	0	0	1
I-0,3	4	36	0	0	1
I-0,3	5	36	0	0	1
I-0,3	1	42	0	0	1
I-0,3	2	42	0	0	1

I-0,3	3	42	0	0	1
I-0,3	4	42	0	0	1
I-0,3	5	42	0	0	1
control	1	1	100	1	0
control	2	1	100	1	0
control	3	1	100	1	0
control	4	1	100	1	0
control	5	1	100	1	0
control	1	2	100	1	0
control	2	2	100	1	0
control	3	2	100	1	0
control	4	2	100	1	0
control	5	2	100	1	0
control	1	3	0	0	0
control	2	3	0	0	0
control	3	3	0	0	0
control	4	3	0	0	0
control	5	3	0	0	0
control	1	6	0	0	0
control	2	6	0	0	0
control	3	6	0	0	0
control	4	6	0	0	0
control	5	6	0	0	0
control	1	9	0	0	0
control	2	9	0	0	0
control	3	9	0	0	0
control	4	9	0	0	0
control	5	9	0	0	0
control	1	12	0	0	0
control	2	12	0	0	0
control	3	12	0	0	0
control	4	12	0	0	0
control	5	12	0	0	0
control	1	15	0	0	1
control	2	15	0	0	1
control	3	15	0	0	1
control	4	15	0	0	1
control	5	15	0	0	1
control	1	18	0	0	1
control	2	18	0	0	1
control	3	18	0	0	1
control	4	18	0	0	1
control	5	18	0	0	1
control	1	21	0	0	1
control	2	21	0	0	1

control	3	21	0	0	1
control	4	21	0	0	1
control	5	21	0	0	1
control	1	24	0	0	1
control	2	24	0	0	1
control	3	24	0	0	1
control	4	24	0	0	1
control	5	24	0	0	1
control	1	27	0	0	1
control	2	27	0	0	1
control	3	27	0	0	1
control	4	27	0	0	1
control	5	27	0	0	1
control	1	30	0	0	1
control	2	30	0	0	1
control	3	30	0	0	1
control	4	30	0	0	1
control	5	30	0	0	1
control	1	33	0	0	1
control	2	33	0	0	1
control	3	33	0	0	1
control	4	33	0	0	1
control	5	33	0	0	1
control	1	36	0	0	1
control	2	36	0	0	1
control	3	36	0	0	1
control	4	36	0	0	1
control	5	36	0	0	1
control	1	42	0	0	1
control	2	42	0	0	1
control	3	42	0	0	1
control	4	42	0	0	1
control	5	42	0	0	1
placebo	1	1	100	1	0
placebo	2	1	100	1	0
placebo	3	1	100	1	0
placebo	4	1	100	1	0
placebo	5	1	100	1	0
placebo	1	2	100	1	0
placebo	2	2	100	1	0
placebo	3	2	100	1	0
placebo	4	2	100	1	0
placebo	5	2	100	1	0
placebo	1	3	25	1	0
placebo	2	3	25	1	0

placebo	3	3	0	0	0
placebo	4	3	0	0	0
placebo	5	3	100	1	0
placebo	1	6	75	1	0
placebo	2	6	75	1	0
placebo	3	6	75	1	0
placebo	4	6	50	1	0
placebo	5	6	100	1	0
placebo	1	9	100	1	0
placebo	2	9	100	1	0
placebo	3	9	100	1	0
placebo	4	9	100	1	0
placebo	5	9	100	1	0
placebo	1	12	100	1	0
placebo	2	12	75	1	0
placebo	3	12	100	1	0
placebo	4	12	100	1	0
placebo	5	12	100	1	0
placebo	1	15	100	1	0
placebo	2	15	100	1	0
placebo	3	15	100	1	0
placebo	4	15	100	1	0
placebo	5	15	100	1	0
placebo	1	18	100	1	0
placebo	2	18	100	1	0
placebo	3	18	100	1	0
placebo	4	18	100	1	0
placebo	5	18	100	1	0
placebo	1	21	100	1	0
placebo	2	21	100	1	0
placebo	3	21	100	1	0
placebo	4	21	100	1	0
placebo	5	21	100	1	0
placebo	1	24	100	1	0
placebo	2	24	100	1	0
placebo	3	24	100	1	0
placebo	4	24	100	1	0
placebo	5	24	100	1	0
placebo	1	27	100	1	0
placebo	2	27	100	1	0
placebo	3	27	100	1	0
placebo	4	27	100	1	0
placebo	5	27	100	1	0
placebo	1	30	100	1	0
placebo	2	30	100	1	0

placebo	3	30	100	1	0
placebo	4	30	100	1	0
placebo	5	30	100	1	0
placebo	1	33	100	1	0
placebo	2	33	50	1	0
placebo	3	33	25	1	0
placebo	4	33	25	1	0
placebo	5	33	25	1	0
placebo	1	36	100	1	0
placebo	2	36	100	1	0
placebo	3	36	100	1	0
placebo	4	36	100	1	0
placebo	5	36	75	1	0
placebo	1	42	100	1	0
placebo	2	42	100	1	0
placebo	3	42	100	1	0
placebo	4	42	100	1	0
placebo	5	42	75	1	0

Field bioassays – bait carried and returned by Atta species

species	treatment	doses	repetition	size	carried	returned
A. laevigata	placebo	10	1	44	100	0
A. laevigata	placebo	10	2	56	100	0
A. laevigata	placebo	10	3	35	100	0
A. laevigata	placebo	10	4	58	100	0
A. laevigata	placebo	10	5	44	100	0
A. laevigata	placebo	10	6	44	100	0
A. laevigata	placebo	10	7	34	100	0
A. laevigata	placebo	10	8	45	100	0
A. laevigata	placebo	10	9	65	100	0
A. laevigata	placebo	10	10	57	100	0
A. laevigata	I-0,05	6	1	46	100	0
A. laevigata	I-0,05	6	2	34	100	0
A. laevigata	I-0,05	6	3	34	100	0
A. laevigata	I-0,05	6	4	66	100	15
A. laevigata	I-0,05	6	5	45	100	0
A. laevigata	I-0,05	6	6	34	100	0
A. laevigata	I-0,05	6	7	47	100	30
A. laevigata	I-0,05	6	8	46	100	0
A. laevigata	I-0,05	6	9	48	100	0
A. laevigata	I-0,05	6	10	33	100	0
A. laevigata	I-0,05	10	1	34	100	20
A. laevigata	I-0,05	10	2	34	100	0
A. laevigata	I-0,05	10	3	55	100	10
A. laevigata	I-0,05	10	4	56	100	15
A. laevigata	I-0,05	10	5	55	100	40
A. laevigata	I-0,05	10	6	46	100	0
A. laevigata	I-0,05	10	7	54	100	0
A. laevigata	I-0,05	10	8	54	100	0
A. laevigata	I-0,05	10	9	33	100	0
A. laevigata	I-0,05	10	10	64	100	0
A. laevigata	I-0,05	12	1	46	100	10
A. laevigata	I-0,05	12	2	55	100	5
A. laevigata	I-0,05	12	3	55	100	0
A. laevigata	I-0,05	12	4	34	100	0
A. laevigata	I-0,05	12	5	45	100	0
A. laevigata	I-0,05	12	6	47	100	10
A. laevigata	I-0,05	12	7	56	100	15
A. laevigata	I-0,05	12	8	33	100	0
A. laevigata	I-0,05	12	9	44	100	0
A. laevigata	I-0,05	12	10	55	100	0
A. laevigata	I-0,1	6	1	57	100	0
A. laevigata	I-0,1	6	2	57	100	10

A. laevigata	I-0,1	6	3	54	100	20
A. laevigata	I-0,1	6	4	45	100	10
A. laevigata	I-0,1	6	5	66	100	30
A. laevigata	I-0,1	6	6	58	100	10
A. laevigata	I-0,1	6	7	44	100	0
A. laevigata	I-0,1	6	8	45	100	0
A. laevigata	I-0,1	6	9	45	100	0
A. laevigata	I-0,1	6	10	44	100	5
A. laevigata	I-0,1	10	1	46	100	15
A. laevigata	I-0,1	10	2	35	100	0
A. laevigata	I-0,1	10	3	48	100	10
A. laevigata	I-0,1	10	4	57	100	0
A. laevigata	I-0,1	10	5	53	100	0
A. laevigata	I-0,1	10	6	36	100	10
A. laevigata	I-0,1	10	7	34	100	0
A. laevigata	I-0,1	10	8	56	100	15
A. laevigata	I-0,1	10	9	67	100	10
A. laevigata	I-0,1	10	10	33	100	0
A. laevigata	I-0,1	12	1	44	100	0
A. laevigata	I-0,1	12	2	35	100	0
A. laevigata	I-0,1	12	3	56	100	5
A. laevigata	I-0,1	12	4	55	0	0
A. laevigata	I-0,1	12	5	56	100	2
A. laevigata	I-0,1	12	6	104	100	2
A. laevigata	I-0,1	12	7	57	100	0
A. laevigata	I-0,1	12	8	56	100	0
A. laevigata	I-0,1	12	9	56	100	0
A. laevigata	I-0,1	12	10	45	100	0
A. laevigata	I-0,2	6	1	55	100	0
A. laevigata	I-0,2	6	2	45	100	0
A. laevigata	I-0,2	6	3	48	100	0
A. laevigata	I-0,2	6	4	34	100	0
A. laevigata	I-0,2	6	5	46	100	0
A. laevigata	I-0,2	6	6	66	100	0
A. laevigata	I-0,2	6	7	56	100	0
A. laevigata	I-0,2	6	8	66	100	0
A. laevigata	I-0,2	6	9	45	100	0
A. laevigata	I-0,2	6	10	59	100	0
A. laevigata	I-0,2	10	1	46	100	0
A. laevigata	I-0,2	10	2	54	100	0
A. laevigata	I-0,2	10	3	94	100	0
A. laevigata	I-0,2	10	4	33	10	0
A. laevigata	I-0,2	10	5	34	100	2
A. laevigata	I-0,2	10	6	44	100	0
A. laevigata	I-0,2	10	7	45	100	0

A. laevigata	I-0,2	10	8	48	100	0
A. laevigata	I-0,2	10	9	57	100	0
A. laevigata	I-0,2	10	10	44	100	0
A. laevigata	I-0,2	12	1	67	100	0
A. laevigata	I-0,2	12	2	44	100	0
A. laevigata	I-0,2	12	3	49	100	2
A. laevigata	I-0,2	12	4	47	100	0
A. laevigata	I-0,2	12	5	57	100	0
A. laevigata	I-0,2	12	6	34	100	0
A. laevigata	I-0,2	12	7	45	100	0
A. laevigata	I-0,2	12	8	34	100	0
A. laevigata	I-0,2	12	9	56	100	0
A. laevigata	I-0,2	12	10	45	100	0
A. laevigata	I-0,3	6	1	45	100	0
A. laevigata	I-0,3	6	2	45	100	0
A. laevigata	I-0,3	6	3	58	100	0
A. laevigata	I-0,3	6	4	56	100	0
A. laevigata	I-0,3	6	5	34	100	0
A. laevigata	I-0,3	6	6	45	100	0
A. laevigata	I-0,3	6	7	56	100	0
A. laevigata	I-0,3	6	8	34	100	0
A. laevigata	I-0,3	6	9	56	100	0
A. laevigata	I-0,3	6	10	75	100	0
A. laevigata	I-0,3	10	1	34	100	0
A. laevigata	I-0,3	10	2	44	100	0
A. laevigata	I-0,3	10	3	55	100	0
A. laevigata	I-0,3	10	4	45	100	0
A. laevigata	I-0,3	10	5	44	100	0
A. laevigata	I-0,3	10	6	33	100	0
A. laevigata	I-0,3	10	7	67	100	0
A. laevigata	I-0,3	10	8	44	100	0
A. laevigata	I-0,3	10	9	52	100	0
A. laevigata	I-0,3	10	10	56	100	0
A. laevigata	I-0,3	12	1	55	100	0
A. laevigata	I-0,3	12	2	57	100	0
A. laevigata	I-0,3	12	3	56	100	0
A. laevigata	I-0,3	12	4	45	100	0
A. laevigata	I-0,3	12	5	34	100	0
A. laevigata	I-0,3	12	6	57	100	0
A. laevigata	I-0,3	12	7	46	100	0
A. laevigata	I-0,3	12	8	55	100	0
A. laevigata	I-0,3	12	9	44	100	0
A. laevigata	I-0,3	12	10	56	100	0
A. laevigata	control	10	1	57	100	30
A. laevigata	control	10	2	57	100	20

A. laevigata	control	10	3	44	100	20
A. laevigata	control	10	4	34	100	65
A. laevigata	control	10	5	44	100	5
A. laevigata	control	10	6	45	100	0
A. laevigata	control	10	7	57	100	0
A. laevigata	control	10	8	45	100	0
A. laevigata	control	10	9	55	100	0
A. laevigata	control	10	10	45	100	0
A. sexdens	placebo	10	1	20	100	0
A. sexdens	placebo	10	2	12	100	0
A. sexdens	placebo	10	3	24	70	0
A. sexdens	placebo	10	4	15	100	0
A. sexdens	placebo	10	5	10	100	0
A. sexdens	placebo	10	6	12	100	0
A. sexdens	placebo	10	7	12	100	0
A. sexdens	placebo	10	8	9	100	5
A. sexdens	placebo	10	9	12	100	0
A. sexdens	placebo	10	10	28	100	0
A. sexdens	I-0,05	6	1	16	100	5
A. sexdens	I-0,05	6	2	12	100	0
A. sexdens	I-0,05	6	3	36	100	0
A. sexdens	I-0,05	6	4	9	100	0
A. sexdens	I-0,05	6	5	16	100	0
A. sexdens	I-0,05	6	6	40	100	0
A. sexdens	I-0,05	6	7	12	100	0
A. sexdens	I-0,05	6	8	12	100	0
A. sexdens	I-0,05	6	9	10	100	0
A. sexdens	I-0,05	6	10	12	100	0
A. sexdens	I-0,05	10	1	30	100	0
A. sexdens	I-0,05	10	2	10	100	0
A. sexdens	I-0,05	10	3	9	100	0
A. sexdens	I-0,05	10	4	10	100	0
A. sexdens	I-0,05	10	5	10	100	0
A. sexdens	I-0,05	10	6	12	100	0
A. sexdens	I-0,05	10	7	12	100	0
A. sexdens	I-0,05	10	8	20	100	0
A. sexdens	I-0,05	10	9	10	100	5
A. sexdens	I-0,05	10	10	10	100	0
A. sexdens	I-0,05	12	1	15	100	0
A. sexdens	I-0,05	12	2	10	100	0
A. sexdens	I-0,05	12	3	28	100	0
A. sexdens	I-0,05	12	4	32	100	10
A. sexdens	I-0,05	12	5	16	100	0
A. sexdens	I-0,05	12	6	28	100	0
A. sexdens	I-0,05	12	7	20	100	0

A. sexdens	I-0,05	12	8	28	100	0
A. sexdens	I-0,05	12	9	15	100	5
A. sexdens	I-0,05	12	10	24	100	5
A. sexdens	I-0,1	6	1	15	100	5
A. sexdens	I-0,1	6	2	30	100	0
A. sexdens	I-0,1	6	3	10	100	0
A. sexdens	I-0,1	6	4	10	100	0
A. sexdens	I-0,1	6	5	12	100	0
A. sexdens	I-0,1	6	6	20	100	0
A. sexdens	I-0,1	6	7	30	100	0
A. sexdens	I-0,1	6	8	20	100	0
A. sexdens	I-0,1	6	9	12	100	0
A. sexdens	I-0,1	6	10	0	100	0
A. sexdens	I-0,1	10	1	24	100	0
A. sexdens	I-0,1	10	2	15	100	0
A. sexdens	I-0,1	10	3	12	100	5
A. sexdens	I-0,1	10	4	9	100	0
A. sexdens	I-0,1	10	5	20	100	5
A. sexdens	I-0,1	10	6	20	100	0
A. sexdens	I-0,1	10	7	12	100	0
A. sexdens	I-0,1	10	8	25	100	0
A. sexdens	I-0,1	10	9	36	100	0
A. sexdens	I-0,1	10	10	16	100	0
A. sexdens	I-0,1	12	1	9	100	0
A. sexdens	I-0,1	12	2	10	100	5
A. sexdens	I-0,1	12	3	12	100	0
A. sexdens	I-0,1	12	4	18	100	0
A. sexdens	I-0,1	12	5	20	100	0
A. sexdens	I-0,1	12	6	9	100	0
A. sexdens	I-0,1	12	7	20	100	0
A. sexdens	I-0,1	12	8	16	100	0
A. sexdens	I-0,1	12	9	12	70	0
A. sexdens	I-0,1	12	10	15	100	0
A. sexdens	I-0,2	6	1	12	100	0
A. sexdens	I-0,2	6	2	15	100	0
A. sexdens	I-0,2	6	3	12	100	0
A. sexdens	I-0,2	6	4	12	100	0
A. sexdens	I-0,2	6	5	18	100	0
A. sexdens	I-0,2	6	6	12	100	0
A. sexdens	I-0,2	6	7	32	100	0
A. sexdens	I-0,2	6	8	15	100	0
A. sexdens	I-0,2	6	9	18	100	0
A. sexdens	I-0,2	6	10	15	100	0
A. sexdens	I-0,2	10	1	12	100	0
A. sexdens	I-0,2	10	2	12	100	0

A. sexdens	I-0,2	10	3	16	100	0
A. sexdens	I-0,2	10	4	12	100	0
A. sexdens	I-0,2	10	5	12	100	0
A. sexdens	I-0,2	10	6	12	100	0
A. sexdens	I-0,2	10	7	25	100	0
A. sexdens	I-0,2	10	8	15	100	0
A. sexdens	I-0,2	10	9	20	100	0
A. sexdens	I-0,2	10	10	16	100	0
A. sexdens	I-0,2	12	1	20	100	0
A. sexdens	I-0,2	12	2	15	100	0
A. sexdens	I-0,2	12	3	24	100	0
A. sexdens	I-0,2	12	4	20	100	0
A. sexdens	I-0,2	12	5	16	100	0
A. sexdens	I-0,2	12	6	9	100	0
A. sexdens	I-0,2	12	7	16	100	0
A. sexdens	I-0,2	12	8	16	100	0
A. sexdens	I-0,2	12	9	16	100	0
A. sexdens	I-0,2	12	10	30	100	0
A. sexdens	I-0,3	6	1	12	100	0
A. sexdens	I-0,3	6	2	12	100	0
A. sexdens	I-0,3	6	3	20	100	0
A. sexdens	I-0,3	6	4	12	100	0
A. sexdens	I-0,3	6	5	25	100	0
A. sexdens	I-0,3	6	6	20	100	0
A. sexdens	I-0,3	6	7	30	100	0
A. sexdens	I-0,3	6	8	20	100	0
A. sexdens	I-0,3	6	9	12	100	0
A. sexdens	I-0,3	6	10	15	100	0
A. sexdens	I-0,3	10	1	12	100	0
A. sexdens	I-0,3	10	2	10	100	0
A. sexdens	I-0,3	10	3	16	100	0
A. sexdens	I-0,3	10	4	20	100	0
A. sexdens	I-0,3	10	5	12	100	0
A. sexdens	I-0,3	10	6	25	100	0
A. sexdens	I-0,3	10	7	12	100	0
A. sexdens	I-0,3	10	8	18	100	0
A. sexdens	I-0,3	10	9	20	100	0
A. sexdens	I-0,3	10	10	20	100	0
A. sexdens	I-0,3	12	1	9	100	0
A. sexdens	I-0,3	12	2	16	100	0
A. sexdens	I-0,3	12	3	15	100	0
A. sexdens	I-0,3	12	4	20	100	0
A. sexdens	I-0,3	12	5	20	100	0
A. sexdens	I-0,3	12	6	24	100	0
A. sexdens	I-0,3	12	7	16	100	0

A. sexdens	I-0,3	12	8	20	75	0
A. sexdens	I-0,3	12	9	28	100	0
A. sexdens	I-0,3	12	10	24	100	0
A. sexdens	control	10	1	16	100	0
A. sexdens	control	10	2	18	100	0
A. sexdens	control	10	3	12	100	0
A. sexdens	control	10	4	24	100	0
A. sexdens	control	10	5	20	100	0
A. sexdens	control	10	6	25	100	0
A. sexdens	control	10	7	24	100	0
A. sexdens	control	10	8	24	100	0
A. sexdens	control	10	9	20	100	0
A. sexdens	control	10	10	12	100	0

Field bioassays – bait carried and returned by Acromyrmex

species	treatment	doses	repetition	size	carried	returned
Ac. lundii	placebo	10	1	1	100	0
Ac. lundii	placebo	10	2	1	100	0
Ac. lundii	placebo	10	3	1	100	0
Ac. lundii	placebo	10	4	1	100	0
Ac. lundii	placebo	10	5	1	100	0
Ac. lundii	placebo	10	6	2	100	0
Ac. lundii	placebo	10	7	1	100	0
Ac. lundii	placebo	10	8	2	100	0
Ac. lundii	placebo	10	9	1	100	0
Ac. lundii	placebo	10	10	1	100	0
Ac. lundii	I-0,05	6	1	1	100	0
Ac. lundii	I-0,05	6	2	1	100	0
Ac. lundii	I-0,05	6	3	1	100	0
Ac. lundii	I-0,05	6	4	1	5	0
Ac. lundii	I-0,05	6	5	1	100	0
Ac. lundii	I-0,05	6	6	1	100	0
Ac. lundii	I-0,05	6	7	1	100	0
Ac. lundii	I-0,05	6	8	1	100	0
Ac. lundii	I-0,05	6	9	1	100	0
Ac. lundii	I-0,05	6	10	1	100	0
Ac. lundii	I-0,05	10	1	1	5	5
Ac. lundii	I-0,05	10	2	1	100	0
Ac. lundii	I-0,05	10	3	1	100	0
Ac. lundii	I-0,05	10	6	2	100	0
Ac. lundii	I-0,05	10	7	1	100	0
Ac. lundii	I-0,05	10	8	1	5	0
Ac. lundii	I-0,05	10	9	1	100	0
Ac. lundii	I-0,05	10	10	1	100	0
Ac. lundii	I-0,05	12	3	1	100	0
Ac. lundii	I-0,05	12	4	1	100	0
Ac. lundii	I-0,05	12	5	1	100	0
Ac. lundii	I-0,05	12	6	1	100	0
Ac. lundii	I-0,05	12	7	1	100	0
Ac. lundii	I-0,05	12	8	1	100	0
Ac. lundii	I-0,05	12	9	1	100	0
Ac. lundii	I-0,05	12	10	1	100	0
Ac. lundii	I-0,1	6	1	1	100	0
Ac. lundii	I-0,1	6	2	1	100	0
Ac. lundii	I-0,1	6	3	1	100	0
Ac. lundii	I-0,1	6	4	1	100	0
Ac. lundii	I-0,1	6	5	1	20	0
Ac. lundii	I-0,1	6	6	1	100	0

Ac. lundii	I-0,1	6	7	1	100	0
Ac. lundii	I-0,1	6	8	1	100	0
Ac. lundii	I-0,1	6	9	1	100	0
Ac. lundii	I-0,1	6	10	1	100	0
Ac. lundii	I-0,1	10	1	1	100	0
Ac. lundii	I-0,1	10	2	1	100	0
Ac. lundii	I-0,1	10	3	1	5	0
Ac. lundii	I-0,1	10	4	1	30	0
Ac. lundii	I-0,1	10	5	1	100	0
Ac. lundii	I-0,1	10	6	1	100	0
Ac. lundii	I-0,1	10	7	1	100	0
Ac. lundii	I-0,1	10	8	1	100	0
Ac. lundii	I-0,1	10	9	1	100	0
Ac. lundii	I-0,1	10	10	1	100	0
Ac. lundii	I-0,1	12	1	1	50	0
Ac. lundii	I-0,1	12	2	1	100	0
Ac. lundii	I-0,1	12	3	1	100	0
Ac. lundii	I-0,1	12	4	1	100	0
Ac. lundii	I-0,1	12	5	1	100	0
Ac. lundii	I-0,1	12	6	1	100	0
Ac. lundii	I-0,1	12	7	1	100	0
Ac. lundii	I-0,1	12	8	1	70	0
Ac. lundii	I-0,1	12	9	1	70	0
Ac. lundii	I-0,1	12	10	1	60	0
Ac. lundii	I-0,2	6	1	1	100	0
Ac. lundii	I-0,2	6	3	1	100	0
Ac. lundii	I-0,2	6	4	1	40	0
Ac. lundii	I-0,2	6	5	1	100	0
Ac. lundii	I-0,2	6	6	1	100	0
Ac. lundii	I-0,2	6	7	1	100	0
Ac. lundii	I-0,2	6	8	1	100	0
Ac. lundii	I-0,2	6	9	1	10	0
Ac. lundii	I-0,2	6	10	1	50	0
Ac. lundii	I-0,2	10	1	1	10	0
Ac. lundii	I-0,2	10	2	1	30	0
Ac. lundii	I-0,2	10	3	1	100	0
Ac. lundii	I-0,2	10	4	1	10	0
Ac. lundii	I-0,2	10	5	1	100	0
Ac. lundii	I-0,2	10	6	1	40	0
Ac. lundii	I-0,2	10	7	1	100	0
Ac. lundii	I-0,2	10	8	1	100	0
Ac. lundii	I-0,2	10	9	1	100	0
Ac. lundii	I-0,2	10	10	1	100	0
Ac. lundii	I-0,2	12	1	1	10	0
Ac. lundii	I-0,2	12	2	1	5	0

Ac. lundii	I-0,2	12	3	1	10	0
Ac. lundii	I-0,2	12	4	1	70	0
Ac. lundii	I-0,2	12	5	1	100	0
Ac. lundii	I-0,2	12	6	1	100	0
Ac. lundii	I-0,2	12	7	1	100	0
Ac. lundii	I-0,2	12	8	1	100	0
Ac. lundii	I-0,2	12	9	1	100	0
Ac. lundii	I-0,2	12	10	1	100	0
Ac. lundii	I-0,3	6	1	1	100	0
Ac. lundii	I-0,3	6	2	1	100	0
Ac. lundii	I-0,3	6	3	1	100	0
Ac. lundii	I-0,3	6	4	1	100	0
Ac. lundii	I-0,3	6	5	1	100	0
Ac. lundii	I-0,3	6	6	1	90	0
Ac. lundii	I-0,3	6	7	1	100	0
Ac. lundii	I-0,3	6	8	1	100	0
Ac. lundii	I-0,3	6	9	1	100	0
Ac. lundii	I-0,3	6	10	1	50	0
Ac. lundii	I-0,3	10	1	1	100	0
Ac. lundii	I-0,3	10	2	1	100	0
Ac. lundii	I-0,3	10	3	1	100	0
Ac. lundii	I-0,3	10	4	1	10	0
Ac. lundii	I-0,3	10	5	1	100	0
Ac. lundii	I-0,3	10	6	1	100	0
Ac. lundii	I-0,3	10	7	1	10	0
Ac. lundii	I-0,3	10	8	1	10	0
Ac. lundii	I-0,3	10	9	1	30	0
Ac. lundii	I-0,3	10	10	1	100	0
Ac. lundii	I-0,3	12	1	1	10	0
Ac. lundii	I-0,3	12	2	1	100	0
Ac. lundii	I-0,3	12	3	1	5	0
Ac. lundii	I-0,3	12	4	1	100	0
Ac. lundii	I-0,3	12	5	1	97	0
Ac. lundii	I-0,3	12	6	1	100	0
Ac. lundii	I-0,3	12	7	1	100	0
Ac. lundii	I-0,3	12	8	1	100	0
Ac. lundii	I-0,3	12	9	1	100	0
Ac. lundii	I-0,3	12	10	1	100	0
Ac. lundii	control	10	1	1	100	0
Ac. lundii	control	10	2	1	100	0
Ac. lundii	control	10	4	1	100	0
Ac. lundii	control	10	5	1	100	0
Ac. lundii	control	10	6	1	100	0
Ac. lundii	control	10	7	1	100	0
Ac. lundii	control	10	8	1	30	0

Ac. lundii	control	10	9	1	100	0
Ac. lundii	control	10	10	1	100	0

Field bioassays - Fig6a and Fig6b

species	treatment	time	activity	censor
laevigata	placebo	120	1	0
laevigata	placebo	120	2	0
laevigata	placebo	120	3	0
laevigata	placebo	120	4	0
laevigata	placebo	120	5	0
laevigata	placebo	120	6	0
laevigata	placebo	120	7	0
laevigata	placebo	120	8	0
laevigata	placebo	120	9	0
laevigata	placebo	120	10	0
laevigata	control	2	1	1
laevigata	control	15	1	1
laevigata	control	15	2	1
laevigata	control	15	3	1
laevigata	control	15	4	1
laevigata	control	15	5	1
laevigata	control	30	1	1
laevigata	control	30	2	1
laevigata	control	120	1	0
laevigata	control	120	2	0
laevigata	I-0.05-6	2	1	1
laevigata	I-0.05-6	30	1	1
laevigata	I-0.05-6	120	1	0
laevigata	I-0.05-6	120	2	0
laevigata	I-0.05-6	120	3	0
laevigata	I-0.05-6	120	4	0
laevigata	I-0.05-6	120	5	0
laevigata	I-0.05-6	120	6	0
laevigata	I-0.05-6	120	7	0
laevigata	I-0.05-6	120	8	0
laevigata	I-0.05-10	2	1	1
laevigata	I-0.05-10	30	1	1
laevigata	I-0.05-10	30	2	1
laevigata	I-0.05-10	120	1	0
laevigata	I-0.05-10	120	2	0
laevigata	I-0.05-10	120	3	0
laevigata	I-0.05-10	120	4	0
laevigata	I-0.05-10	120	5	0
laevigata	I-0.05-10	120	6	0
laevigata	I-0.05-10	120	7	0
laevigata	I-0.05-12	90	1	1
laevigata	I-0.05-12	120	1	1

laevigata	I-0.05-12	120	1	0
laevigata	I-0.05-12	120	2	0
laevigata	I-0.05-12	120	3	0
laevigata	I-0.05-12	120	4	0
laevigata	I-0.05-12	120	5	0
laevigata	I-0.05-12	120	6	0
laevigata	I-0.05-12	120	7	0
laevigata	I-0.05-12	120	8	0
laevigata	I-0.1-6	7	1	1
laevigata	I-0.1-6	30	1	1
laevigata	I-0.1-6	90	1	1
laevigata	I-0.1-6	120	1	0
laevigata	I-0.1-6	120	2	0
laevigata	I-0.1-6	120	3	0
laevigata	I-0.1-6	120	4	0
laevigata	I-0.1-6	120	5	0
laevigata	I-0.1-6	120	6	0
laevigata	I-0.1-6	120	7	0
laevigata	I-0.1-10	2	1	1
laevigata	I-0.1-10	2	2	1
laevigata	I-0.1-10	2	3	1
laevigata	I-0.1-10	2	4	1
laevigata	I-0.1-10	2	5	1
laevigata	I-0.1-10	30	1	1
laevigata	I-0.1-10	120	1	0
laevigata	I-0.1-10	120	2	0
laevigata	I-0.1-10	120	3	0
laevigata	I-0.1-10	120	4	0
laevigata	I-0.1-12	7	1	1
laevigata	I-0.1-12	7	2	1
laevigata	I-0.1-12	15	1	1
laevigata	I-0.1-12	15	2	1
laevigata	I-0.1-12	30	1	1
laevigata	I-0.1-12	120	1	0
laevigata	I-0.1-12	120	2	0
laevigata	I-0.1-12	120	3	0
laevigata	I-0.1-12	120	4	0
laevigata	I-0.1-12	120	5	0
laevigata	I-0.2-6	2	1	1
laevigata	I-0.2-6	2	2	1
laevigata	I-0.2-6	2	3	1
laevigata	I-0.2-6	2	4	1
laevigata	I-0.2-6	15	1	1
laevigata	I-0.2-6	15	2	1
laevigata	I-0.2-6	15	3	1

laevigata	I-0.2-6	15	4	1
laevigata	I-0.2-6	30	1	1
laevigata	I-0.2-6	120	1	1
laevigata	I-0.2-10	2	1	1
laevigata	I-0.2-10	2	2	1
laevigata	I-0.2-10	7	1	1
laevigata	I-0.2-10	7	2	1
laevigata	I-0.2-10	7	3	1
laevigata	I-0.2-10	15	1	1
laevigata	I-0.2-10	15	2	1
laevigata	I-0.2-10	15	3	1
laevigata	I-0.2-10	15	4	1
laevigata	I-0.2-10	120	1	0
laevigata	I-0.2-12	7	1	1
laevigata	I-0.2-12	7	2	1
laevigata	I-0.2-12	15	1	1
laevigata	I-0.2-12	15	2	1
laevigata	I-0.2-12	15	3	1
laevigata	I-0.2-12	15	4	1
laevigata	I-0.2-12	30	1	1
laevigata	I-0.2-12	30	2	1
laevigata	I-0.2-12	90	1	1
laevigata	I-0.2-12	120	1	0
laevigata	I-0.3-6	7	1	1
laevigata	I-0.3-6	7	2	1
laevigata	I-0.3-6	15	1	1
laevigata	I-0.3-6	15	2	1
laevigata	I-0.3-6	30	1	1
laevigata	I-0.3-6	30	2	1
laevigata	I-0.3-6	90	1	1
laevigata	I-0.3-6	120	1	0
laevigata	I-0.3-6	120	2	0
laevigata	I-0.3-6	120	3	0
laevigata	I-0.3-10	2	1	1
laevigata	I-0.3-10	7	1	1
laevigata	I-0.3-10	15	1	1
laevigata	I-0.3-10	15	2	1
laevigata	I-0.3-10	30	1	1
laevigata	I-0.3-10	60	1	1
laevigata	I-0.3-10	60	2	1
laevigata	I-0.3-10	120	1	0
laevigata	I-0.3-10	120	2	0
laevigata	I-0.3-10	120	3	0
laevigata	I-0.3-12	2	1	1
laevigata	I-0.3-12	2	2	1

laevigata	I-0.3-12	15	1	1
laevigata	I-0.3-12	15	2	1
laevigata	I-0.3-12	30	1	1
laevigata	I-0.3-12	30	2	1
laevigata	I-0.3-12	30	3	1
laevigata	I-0.3-12	60	1	1
laevigata	I-0.3-12	60	2	1
laevigata	I-0.3-12	120	1	0
sexdens	placebo	15	1	1
sexdens	placebo	150	2	1
sexdens	placebo	30	1	1
sexdens	placebo	30	2	1
sexdens	placebo	120	1	0
sexdens	placebo	120	2	0
sexdens	placebo	120	3	0
sexdens	placebo	120	4	0
sexdens	placebo	120	5	0
sexdens	placebo	120	6	0
sexdens	control	2	1	1
sexdens	control	2	2	1
sexdens	control	2	3	1
sexdens	control	2	4	1
sexdens	control	2	5	1
sexdens	control	2	6	1
sexdens	control	2	7	1
sexdens	control	15	1	1
sexdens	control	15	2	1
sexdens	control	30	1	1
sexdens	I-0.05-6	2	1	1
sexdens	I-0.05-6	2	2	1
sexdens	I-0.05-6	15	1	1
sexdens	I-0.05-6	30	1	1
sexdens	I-0.05-6	120	1	0
sexdens	I-0.05-6	120	2	0
sexdens	I-0.05-6	120	3	0
sexdens	I-0.05-6	120	4	0
sexdens	I-0.05-6	120	5	0
sexdens	I-0.05-6	120	6	0
sexdens	I-0.05-10	2	1	1
sexdens	I-0.05-10	2	2	1
sexdens	I-0.05-10	15	1	1
sexdens	I-0.05-10	30	1	1
sexdens	I-0.05-10	120	1	0
sexdens	I-0.05-10	120	2	0
sexdens	I-0.05-10	120	3	0

sexdens	I-0.05-10	120	4	0
sexdens	I-0.05-10	120	5	0
sexdens	I-0.05-10	120	6	0
sexdens	I-0.05-12	15	1	1
sexdens	I-0.05-12	15	2	1
sexdens	I-0.05-12	15	3	1
sexdens	I-0.05-12	15	4	1
sexdens	I-0.05-12	15	5	1
sexdens	I-0.05-12	30	1	1
sexdens	I-0.05-12	30	2	1
sexdens	I-0.05-12	30	3	1
sexdens	I-0.05-12	30	4	1
sexdens	I-0.05-12	30	5	1
sexdens	I-0.1-6	15	1	1
sexdens	I-0.1-6	30	1	1
sexdens	I-0.1-6	30	2	1
sexdens	I-0.1-6	30	3	1
sexdens	I-0.1-6	30	4	1
sexdens	I-0.1-6	30	5	1
sexdens	I-0.1-6	30	6	1
sexdens	I-0.1-6	30	7	1
sexdens	I-0.1-6	30	8	1
sexdens	I-0.1-6	30	9	1
sexdens	I-0.1-10	2	1	1
sexdens	I-0.1-10	2	2	1
sexdens	I-0.1-10	2	3	1
sexdens	I-0.1-10	2	4	1
sexdens	I-0.1-10	2	5	1
sexdens	I-0.1-10	2	6	1
sexdens	I-0.1-10	7	1	1
sexdens	I-0.1-10	7	2	1
sexdens	I-0.1-10	30	1	1
sexdens	I-0.1-10	30	2	1
sexdens	I-0.1-12	2	1	1
sexdens	I-0.1-12	2	2	1
sexdens	I-0.1-12	2	3	1
sexdens	I-0.1-12	2	4	1
sexdens	I-0.1-12	2	5	1
sexdens	I-0.1-12	2	6	1
sexdens	I-0.1-12	3	1	1
sexdens	I-0.1-12	3	2	1
sexdens	I-0.1-12	30	1	1
sexdens	I-0.1-12	30	2	1
sexdens	I-0.2-6	2	1	1
sexdens	I-0.2-6	2	2	1

sexdens	I-0.2-6	2	3	1
sexdens	I-0.2-6	2	4	1
sexdens	I-0.2-6	2	5	1
sexdens	I-0.2-6	15	1	1
sexdens	I-0.2-6	15	2	1
sexdens	I-0.2-6	15	3	1
sexdens	I-0.2-6	30	1	1
sexdens	I-0.2-6	60	1	1
sexdens	I-0.2-10	2	1	1
sexdens	I-0.2-10	2	2	1
sexdens	I-0.2-10	2	3	1
sexdens	I-0.2-10	2	4	1
sexdens	I-0.2-10	2	5	1
sexdens	I-0.2-10	2	6	1
sexdens	I-0.2-10	2	7	1
sexdens	I-0.2-10	7	1	1
sexdens	I-0.2-10	7	2	1
sexdens	I-0.2-10	15	1	1
sexdens	I-0.2-12	2	1	1
sexdens	I-0.2-12	2	2	1
sexdens	I-0.2-12	2	3	1
sexdens	I-0.2-12	2	4	1
sexdens	I-0.2-12	15	1	1
sexdens	I-0.2-12	15	2	1
sexdens	I-0.2-12	15	3	1
sexdens	I-0.2-12	15	4	1
sexdens	I-0.2-12	15	5	1
sexdens	I-0.2-12	15	6	1
sexdens	I-0.3-6	2	1	1
sexdens	I-0.3-6	2	2	1
sexdens	I-0.3-6	2	3	1
sexdens	I-0.3-6	2	4	1
sexdens	I-0.3-6	2	5	1
sexdens	I-0.3-6	2	6	1
sexdens	I-0.3-6	2	7	1
sexdens	I-0.3-6	2	8	1
sexdens	I-0.3-6	2	9	1
sexdens	I-0.3-6	15	1	1
sexdens	I-0.3-10	2	1	1
sexdens	I-0.3-10	2	2	1
sexdens	I-0.3-10	2	3	1
sexdens	I-0.3-10	2	4	1
sexdens	I-0.3-10	2	5	1
sexdens	I-0.3-10	3	1	1
sexdens	I-0.3-10	15	1	1

sexdens	I-0.3-10	60	1	1
sexdens	I-0.3-10	60	2	1
sexdens	I-0.3-10	60	3	1
sexdens	I-0.3-12	2	1	1
sexdens	I-0.3-12	2	2	1
sexdens	I-0.3-12	2	3	1
sexdens	I-0.3-12	2	4	1
sexdens	I-0.3-12	2	5	1
sexdens	I-0.3-12	2	6	1
sexdens	I-0.3-12	15	1	1
sexdens	I-0.3-12	15	2	1
sexdens	I-0.3-12	30	1	1
sexdens	I-0.3-12	30	2	1

Field bioassays - Fig6c

species	treatment	time	activity	censor
lundi	placebo	90	1	1
lundi	placebo	90	1	0
lundi	placebo	90	2	0
lundi	placebo	90	3	0
lundi	placebo	90	4	0
lundi	placebo	90	5	0
lundi	placebo	90	6	0
lundi	placebo	90	7	0
lundi	placebo	90	8	0
lundi	placebo	90	9	0
lundi	control	2	1	1
lundi	control	2	2	1
lundi	control	2	3	1
lundi	control	2	4	1
lundi	control	2	5	1
lundi	control	2	6	1
lundi	control	2	7	1
lundi	control	2	8	1
lundi	control	2	9	1
lundi	control	90	1	0
lundi	l-0.05-6	2	1	1
lundi	l-0.05-6	2	2	1
lundi	l-0.05-6	2	3	1
lundi	l-0.05-6	2	4	1
lundi	l-0.05-6	2	5	1
lundi	l-0.05-6	2	6	1
lundi	l-0.05-6	90	1	0
lundi	l-0.05-6	90	2	0
lundi	l-0.05-6	90	3	0
lundi	l-0.05-6	90	4	0
lundi	l-0.05-10	2	1	1
lundi	l-0.05-10	2	2	1
lundi	l-0.05-10	2	3	1
lundi	l-0.05-10	2	4	1
lundi	l-0.05-10	2	5	1
lundi	l-0.05-10	2	6	1
lundi	l-0.05-10	2	7	1
lundi	l-0.05-10	2	8	1
lundi	l-0.05-10	90	1	0
lundi	l-0.05-10	90	1	0
lundi	l-0.05-12	2	1	1
lundi	l-0.05-12	2	2	1

lundi	I-0.05-12	2	3	1
lundi	I-0.05-12	2	4	1
lundi	I-0.05-12	2	5	1
lundi	I-0.05-12	2	6	1
lundi	I-0.05-12	2	7	1
lundi	I-0.05-12	2	8	1
lundi	I-0.05-12	2	9	1
lundi	I-0.05-12	7	1	1
lundi	I-0.1-6	2	1	1
lundi	I-0.1-6	2	2	1
lundi	I-0.1-6	2	3	1
lundi	I-0.1-6	2	4	1
lundi	I-0.1-6	2	5	1
lundi	I-0.1-6	2	6	1
lundi	I-0.1-6	2	7	1
lundi	I-0.1-6	2	8	1
lundi	I-0.1-6	2	9	1
lundi	I-0.1-6	2	10	1
lundi	I-0.1-10	2	1	1
lundi	I-0.1-10	2	2	1
lundi	I-0.1-10	2	3	1
lundi	I-0.1-10	2	4	1
lundi	I-0.1-10	2	5	1
lundi	I-0.1-10	2	6	1
lundi	I-0.1-10	2	7	1
lundi	I-0.1-10	2	8	1
lundi	I-0.1-10	2	9	1
lundi	I-0.1-10	2	10	1
lundi	I-0.1-12	2	1	1
lundi	I-0.1-12	2	2	1
lundi	I-0.1-12	2	3	1
lundi	I-0.1-12	2	4	1
lundi	I-0.1-12	2	5	1
lundi	I-0.1-12	2	6	1
lundi	I-0.1-12	2	7	1
lundi	I-0.1-12	2	8	1
lundi	I-0.1-12	2	9	1
lundi	I-0.1-12	2	10	1
lundi	I-0.2-6	2	1	1
lundi	I-0.2-6	2	2	1
lundi	I-0.2-6	2	3	1
lundi	I-0.2-6	2	4	1
lundi	I-0.2-6	2	5	1
lundi	I-0.2-6	2	6	1
lundi	I-0.2-6	2	7	1

lundi	I-0.2-6	2	8	1
lundi	I-0.2-6	2	9	1
lundi	I-0.2-6	3	10	1
lundi	I-0.2-10	2	1	1
lundi	I-0.2-10	2	2	1
lundi	I-0.2-10	2	3	1
lundi	I-0.2-10	2	4	1
lundi	I-0.2-10	2	5	1
lundi	I-0.2-10	2	6	1
lundi	I-0.2-10	2	7	1
lundi	I-0.2-10	2	8	1
lundi	I-0.2-10	2	9	1
lundi	I-0.2-10	3	10	1
lundi	I-0.2-12	2	1	1
lundi	I-0.2-12	2	2	1
lundi	I-0.2-12	2	3	1
lundi	I-0.2-12	2	4	1
lundi	I-0.2-12	2	5	1
lundi	I-0.2-12	2	6	1
lundi	I-0.2-12	2	7	1
lundi	I-0.2-12	2	8	1
lundi	I-0.2-12	2	9	1
lundi	I-0.2-12	2	10	1
lundi	I-0.3-6	2	1	1
lundi	I-0.3-6	2	2	1
lundi	I-0.3-6	2	3	1
lundi	I-0.3-6	2	4	1
lundi	I-0.3-6	2	5	1
lundi	I-0.3-6	2	6	1
lundi	I-0.3-6	2	7	1
lundi	I-0.3-6	2	8	1
lundi	I-0.3-6	2	9	1
lundi	I-0.3-6	7	1	1
lundi	I-0.3-10	2	1	1
lundi	I-0.3-10	2	2	1
lundi	I-0.3-10	2	3	1
lundi	I-0.3-10	2	4	1
lundi	I-0.3-10	2	5	1
lundi	I-0.3-10	2	6	1
lundi	I-0.3-10	2	7	1
lundi	I-0.3-10	2	8	1
lundi	I-0.3-10	2	9	1
lundi	I-0.3-10	7	1	1
lundi	I-0.3-12	2	1	1
lundi	I-0.3-12	2	2	1

lundi	I-0.3-12	2	3	1
lundi	I-0.3-12	2	4	1
lundi	I-0.3-12	2	5	1
lundi	I-0.3-12	2	6	1
lundi	I-0.3-12	2	7	1
lundi	I-0.3-12	2	8	1
lundi	I-0.3-12	2	9	1
lundi	I-0.3-12	90	1	0

Field bioassays - Fig 7 – Atta

species	treatment	time	mortality	censor
laevigata	placebo	150	1	0
laevigata	placebo	150	2	0
laevigata	placebo	150	3	0
laevigata	placebo	150	4	0
laevigata	placebo	150	5	0
laevigata	placebo	150	6	0
laevigata	placebo	150	7	0
laevigata	placebo	150	8	0
laevigata	placebo	150	9	0
laevigata	placebo	150	10	0
laevigata	control	2	1	1
laevigata	control	30	1	1
laevigata	control	30	2	1
laevigata	control	30	3	1
laevigata	control	30	4	1
laevigata	control	30	5	1
laevigata	control	30	6	1
laevigata	control	150	1	0
laevigata	control	150	2	0
laevigata	control	150	3	0
laevigata	I-0.05-6	30	1	1
laevigata	I-0.05-6	150	1	1
laevigata	I-0.05-6	150	2	0
laevigata	I-0.05-6	150	3	0
laevigata	I-0.05-6	150	4	0
laevigata	I-0.05-6	150	5	0
laevigata	I-0.05-6	150	6	0
laevigata	I-0.05-6	150	7	0
laevigata	I-0.05-6	150	8	0
laevigata	I-0.05-6	150	9	0
laevigata	I-0.05-10	7	1	1
laevigata	I-0.05-10	150	1	1
laevigata	I-0.05-10	150	1	0
laevigata	I-0.05-10	150	2	0
laevigata	I-0.05-10	150	3	0
laevigata	I-0.05-10	150	4	0
laevigata	I-0.05-10	150	5	0
laevigata	I-0.05-10	150	6	0
laevigata	I-0.05-10	150	7	0
laevigata	I-0.05-10	150	8	0
laevigata	I-0.05-12	30	1	1
laevigata	I-0.05-12	30	2	1

laevigata	I-0.05-12	150	1	0
laevigata	I-0.05-12	150	2	0
laevigata	I-0.05-12	150	3	0
laevigata	I-0.05-12	150	4	0
laevigata	I-0.05-12	150	5	0
laevigata	I-0.05-12	150	6	0
laevigata	I-0.05-12	150	7	0
laevigata	I-0.05-12	150	8	0
laevigata	I-0.1-6	90	1	1
laevigata	I-0.1-6	150	1	0
laevigata	I-0.1-6	150	2	0
laevigata	I-0.1-6	150	3	0
laevigata	I-0.1-6	150	4	0
laevigata	I-0.1-6	150	5	0
laevigata	I-0.1-6	150	6	0
laevigata	I-0.1-6	150	7	0
laevigata	I-0.1-6	150	8	0
laevigata	I-0.1-6	150	9	0
laevigata	I-0.1-10	3	1	1
laevigata	I-0.1-10	7	1	1
laevigata	I-0.1-10	7	2	1
laevigata	I-0.1-10	150	1	0
laevigata	I-0.1-10	150	2	0
laevigata	I-0.1-10	150	3	0
laevigata	I-0.1-10	150	4	0
laevigata	I-0.1-10	150	5	0
laevigata	I-0.1-10	150	6	0
laevigata	I-0.1-10	150	7	0
laevigata	I-0.1-12	15	1	1
laevigata	I-0.1-12	15	2	1
laevigata	I-0.1-12	30	1	1
laevigata	I-0.1-12	90	1	1
laevigata	I-0.1-12	150	1	0
laevigata	I-0.1-12	150	2	0
laevigata	I-0.1-12	150	3	0
laevigata	I-0.1-12	150	4	0
laevigata	I-0.1-12	150	5	0
laevigata	I-0.1-12	150	6	0
laevigata	I-0.2-6	7	1	1
laevigata	I-0.2-6	7	2	1
laevigata	I-0.2-6	7	3	1
laevigata	I-0.2-6	15	1	1
laevigata	I-0.2-6	15	2	1
laevigata	I-0.2-6	15	3	1
laevigata	I-0.2-6	15	4	1

laevigata	I-0.2-6	15	5	1
laevigata	I-0.2-6	60	1	1
laevigata	I-0.2-6	150	1	0
laevigata	I-0.2-10	2	1	1
laevigata	I-0.2-10	2	2	1
laevigata	I-0.2-10	15	1	1
laevigata	I-0.2-10	30	1	1
laevigata	I-0.2-10	30	1	1
laevigata	I-0.2-10	60	1	1
laevigata	I-0.2-10	60	1	1
laevigata	I-0.2-10	150	1	0
laevigata	I-0.2-10	150	2	0
laevigata	I-0.2-10	150	3	0
laevigata	I-0.2-12	7	1	1
laevigata	I-0.2-12	7	2	1
laevigata	I-0.2-12	15	1	1
laevigata	I-0.2-12	30	1	1
laevigata	I-0.2-12	30	2	1
laevigata	I-0.2-12	30	3	1
laevigata	I-0.2-12	30	4	1
laevigata	I-0.2-12	60	1	1
laevigata	I-0.2-12	120	1	1
laevigata	I-0.2-12	150	1	0
laevigata	I-0.3-6	7	1	1
laevigata	I-0.3-6	30	1	1
laevigata	I-0.3-6	30	2	1
laevigata	I-0.3-6	30	3	1
laevigata	I-0.3-6	90	1	1
laevigata	I-0.3-6	150	1	0
laevigata	I-0.3-6	150	2	0
laevigata	I-0.3-6	150	3	0
laevigata	I-0.3-6	150	4	0
laevigata	I-0.3-6	150	5	0
laevigata	I-0.3-10	7	1	1
laevigata	I-0.3-10	7	2	1
laevigata	I-0.3-10	7	3	1
laevigata	I-0.3-10	15	1	1
laevigata	I-0.3-10	15	2	1
laevigata	I-0.3-10	30	1	1
laevigata	I-0.3-10	150	1	0
laevigata	I-0.3-10	150	2	0
laevigata	I-0.3-10	150	3	0
laevigata	I-0.3-10	150	4	0
laevigata	I-0.3-12	7	1	1
laevigata	I-0.3-12	7	2	1

laevigata	I-0.3-12	7	3	1
laevigata	I-0.3-12	7	4	1
laevigata	I-0.3-12	30	1	1
laevigata	I-0.3-12	30	2	1
laevigata	I-0.3-12	30	3	1
laevigata	I-0.3-12	60	1	1
laevigata	I-0.3-12	90	1	1
laevigata	I-0.3-12	150	1	0
sexdens	placebo	150	1	0
sexdens	placebo	150	2	0
sexdens	placebo	150	3	0
sexdens	placebo	150	4	0
sexdens	placebo	150	5	0
sexdens	placebo	150	6	0
sexdens	placebo	150	7	0
sexdens	placebo	150	8	0
sexdens	placebo	150	9	0
sexdens	placebo	150	10	0
sexdens	control	30	1	1
sexdens	control	60	1	1
sexdens	control	60	2	1
sexdens	control	60	3	1
sexdens	control	60	4	1
sexdens	control	60	5	1
sexdens	control	60	6	1
sexdens	control	60	7	1
sexdens	control	60	8	1
sexdens	control	150	1	0
sexdens	I-0.05-6	150	1	0
sexdens	I-0.05-6	150	2	0
sexdens	I-0.05-6	150	3	0
sexdens	I-0.05-6	150	4	0
sexdens	I-0.05-6	150	5	0
sexdens	I-0.05-6	150	6	0
sexdens	I-0.05-6	150	7	0
sexdens	I-0.05-6	150	8	0
sexdens	I-0.05-6	150	9	0
sexdens	I-0.05-6	150	10	0
sexdens	I-0.05-10	150	1	0
sexdens	I-0.05-10	150	2	0
sexdens	I-0.05-10	150	3	0
sexdens	I-0.05-10	150	4	0
sexdens	I-0.05-10	150	5	0
sexdens	I-0.05-10	150	6	0
sexdens	I-0.05-10	150	7	0

sexdens	I-0.05-10	150	8	0
sexdens	I-0.05-10	150	9	0
sexdens	I-0.05-10	150	10	0
sexdens	I-0.05-12	150	1	0
sexdens	I-0.05-12	150	2	0
sexdens	I-0.05-12	150	3	0
sexdens	I-0.05-12	150	4	0
sexdens	I-0.05-12	150	5	0
sexdens	I-0.05-12	150	6	0
sexdens	I-0.05-12	150	7	0
sexdens	I-0.05-12	150	8	0
sexdens	I-0.05-12	150	9	0
sexdens	I-0.05-12	150	10	0
sexdens	I-0.1-6	90	1	1
sexdens	I-0.1-6	150	1	0
sexdens	I-0.1-6	150	2	0
sexdens	I-0.1-6	150	3	0
sexdens	I-0.1-6	150	4	0
sexdens	I-0.1-6	150	5	0
sexdens	I-0.1-6	150	6	0
sexdens	I-0.1-6	150	7	0
sexdens	I-0.1-6	150	8	0
sexdens	I-0.1-6	150	9	0
sexdens	I-0.1-10	30	1	1
sexdens	I-0.1-10	30	2	1
sexdens	I-0.1-10	60	1	1
sexdens	I-0.1-10	60	2	1
sexdens	I-0.1-10	60	3	1
sexdens	I-0.1-10	60	4	1
sexdens	I-0.1-10	150	1	1
sexdens	I-0.1-10	150	1	0
sexdens	I-0.1-10	150	2	0
sexdens	I-0.1-10	150	3	0
sexdens	I-0.1-12	30	1	1
sexdens	I-0.1-12	30	2	1
sexdens	I-0.1-12	60	1	1
sexdens	I-0.1-12	60	2	1
sexdens	I-0.1-12	60	3	1
sexdens	I-0.1-12	60	4	1
sexdens	I-0.1-12	60	5	1
sexdens	I-0.1-12	90	1	1
sexdens	I-0.1-12	150	1	0
sexdens	I-0.1-12	150	2	0
sexdens	I-0.2-6	30	1	1
sexdens	I-0.2-6	60	1	1

sexdens	I-0.2-6	60	2	1
sexdens	I-0.2-6	60	3	1
sexdens	I-0.2-6	60	4	1
sexdens	I-0.2-6	60	5	1
sexdens	I-0.2-6	60	6	1
sexdens	I-0.2-6	60	7	1
sexdens	I-0.2-6	150	1	0
sexdens	I-0.2-6	150	2	0
sexdens	I-0.2-10	60	1	1
sexdens	I-0.2-10	60	2	1
sexdens	I-0.2-10	60	3	1
sexdens	I-0.2-10	60	4	1
sexdens	I-0.2-10	60	5	1
sexdens	I-0.2-10	60	6	1
sexdens	I-0.2-10	60	7	1
sexdens	I-0.2-10	60	8	1
sexdens	I-0.2-10	150	1	0
sexdens	I-0.2-10	150	2	0
sexdens	I-0.2-12	60	1	1
sexdens	I-0.2-12	60	2	1
sexdens	I-0.2-12	60	3	1
sexdens	I-0.2-12	60	4	1
sexdens	I-0.2-12	60	5	1
sexdens	I-0.2-12	60	6	1
sexdens	I-0.2-12	60	7	1
sexdens	I-0.2-12	60	8	1
sexdens	I-0.2-12	150	1	0
sexdens	I-0.2-12	150	2	0
sexdens	I-0.3-6	60	1	1
sexdens	I-0.3-6	60	2	1
sexdens	I-0.3-6	60	3	1
sexdens	I-0.3-6	60	4	1
sexdens	I-0.3-6	60	5	1
sexdens	I-0.3-6	60	6	1
sexdens	I-0.3-6	60	7	1
sexdens	I-0.3-6	60	8	1
sexdens	I-0.3-6	150	1	0
sexdens	I-0.3-6	150	2	0
sexdens	I-0.3-10	60	1	1
sexdens	I-0.3-10	60	2	1
sexdens	I-0.3-10	60	3	1
sexdens	I-0.3-10	60	4	1
sexdens	I-0.3-10	60	5	1
sexdens	I-0.3-10	60	6	1
sexdens	I-0.3-10	60	7	1

sexdens	I-0.3-10	60	8	1
sexdens	I-0.3-10	150	1	0
sexdens	I-0.3-10	150	2	0
sexdens	I-0.3-12	60	1	1
sexdens	I-0.3-12	60	2	1
sexdens	I-0.3-12	60	3	1
sexdens	I-0.3-12	60	4	1
sexdens	I-0.3-12	60	5	1
sexdens	I-0.3-12	60	6	1
sexdens	I-0.3-12	60	7	1
sexdens	I-0.3-12	60	8	1
sexdens	I-0.3-12	150	1	0
sexdens	I-0.3-12	150	2	0

Field bioassays - Fig 7 – Acromyrmex

species	treatment	time	mortality	censor
lundi	placebo	90	1	1
lundi	placebo	90	1	0
lundi	placebo	90	2	0
lundi	placebo	90	3	0
lundi	placebo	90	4	0
lundi	placebo	90	5	0
lundi	placebo	90	6	0
lundi	placebo	90	7	0
lundi	placebo	90	8	0
lundi	placebo	90	9	0
lundi	control	2	1	1
lundi	control	2	2	1
lundi	control	2	3	1
lundi	control	2	4	1
lundi	control	2	5	1
lundi	control	2	6	1
lundi	control	2	7	1
lundi	control	2	8	1
lundi	control	2	9	1
lundi	control	90	1	0
lundi	I-0.05-6	2	1	1
lundi	I-0.05-6	2	2	1
lundi	I-0.05-6	2	3	1
lundi	I-0.05-6	2	4	1
lundi	I-0.05-6	2	5	1
lundi	I-0.05-6	2	6	1
lundi	I-0.05-6	90	1	0
lundi	I-0.05-6	90	2	0
lundi	I-0.05-6	90	3	0
lundi	I-0.05-6	90	4	0
lundi	I-0.05-10	2	1	1
lundi	I-0.05-10	2	2	1
lundi	I-0.05-10	2	3	1
lundi	I-0.05-10	2	4	1
lundi	I-0.05-10	2	5	1
lundi	I-0.05-10	2	6	1
lundi	I-0.05-10	2	7	1
lundi	I-0.05-10	2	8	1
lundi	I-0.05-10	90	1	0
lundi	I-0.05-10	90	1	0
lundi	I-0.05-12	2	1	1
lundi	I-0.05-12	2	2	1

lundi	I-0.05-12	2	3	1
lundi	I-0.05-12	2	4	1
lundi	I-0.05-12	2	5	1
lundi	I-0.05-12	2	6	1
lundi	I-0.05-12	2	7	1
lundi	I-0.05-12	2	8	1
lundi	I-0.05-12	2	9	1
lundi	I-0.05-12	7	1	1
lundi	I-0.1-6	2	1	1
lundi	I-0.1-6	2	2	1
lundi	I-0.1-6	2	3	1
lundi	I-0.1-6	2	4	1
lundi	I-0.1-6	2	5	1
lundi	I-0.1-6	2	6	1
lundi	I-0.1-6	2	7	1
lundi	I-0.1-6	2	8	1
lundi	I-0.1-6	2	9	1
lundi	I-0.1-6	2	10	1
lundi	I-0.1-10	2	1	1
lundi	I-0.1-10	2	2	1
lundi	I-0.1-10	2	3	1
lundi	I-0.1-10	2	4	1
lundi	I-0.1-10	2	5	1
lundi	I-0.1-10	2	6	1
lundi	I-0.1-10	2	7	1
lundi	I-0.1-10	2	8	1
lundi	I-0.1-10	2	9	1
lundi	I-0.1-10	2	10	1
lundi	I-0.1-12	2	1	1
lundi	I-0.1-12	2	2	1
lundi	I-0.1-12	2	3	1
lundi	I-0.1-12	2	4	1
lundi	I-0.1-12	2	5	1
lundi	I-0.1-12	2	6	1
lundi	I-0.1-12	2	7	1
lundi	I-0.1-12	2	8	1
lundi	I-0.1-12	2	9	1
lundi	I-0.1-12	2	10	1
lundi	I-0.2-6	2	1	1
lundi	I-0.2-6	2	2	1
lundi	I-0.2-6	2	3	1
lundi	I-0.2-6	2	4	1
lundi	I-0.2-6	2	5	1
lundi	I-0.2-6	2	6	1
lundi	I-0.2-6	2	7	1

lundi	I-0.2-6	2	8	1
lundi	I-0.2-6	2	9	1
lundi	I-0.2-6	3	10	1
lundi	I-0.2-10	2	1	1
lundi	I-0.2-10	2	2	1
lundi	I-0.2-10	2	3	1
lundi	I-0.2-10	2	4	1
lundi	I-0.2-10	2	5	1
lundi	I-0.2-10	2	6	1
lundi	I-0.2-10	2	7	1
lundi	I-0.2-10	2	8	1
lundi	I-0.2-10	2	9	1
lundi	I-0.2-10	3	10	1
lundi	I-0.2-12	2	1	1
lundi	I-0.2-12	2	2	1
lundi	I-0.2-12	2	3	1
lundi	I-0.2-12	2	4	1
lundi	I-0.2-12	2	5	1
lundi	I-0.2-12	2	6	1
lundi	I-0.2-12	2	7	1
lundi	I-0.2-12	2	8	1
lundi	I-0.2-12	2	9	1
lundi	I-0.2-12	2	10	1
lundi	I-0.3-6	2	1	1
lundi	I-0.3-6	2	2	1
lundi	I-0.3-6	2	3	1
lundi	I-0.3-6	2	4	1
lundi	I-0.3-6	2	5	1
lundi	I-0.3-6	2	6	1
lundi	I-0.3-6	2	7	1
lundi	I-0.3-6	2	8	1
lundi	I-0.3-6	2	9	1
lundi	I-0.3-6	7	1	1
lundi	I-0.3-10	2	1	1
lundi	I-0.3-10	2	2	1
lundi	I-0.3-10	2	3	1
lundi	I-0.3-10	2	4	1
lundi	I-0.3-10	2	5	1
lundi	I-0.3-10	2	6	1
lundi	I-0.3-10	2	7	1
lundi	I-0.3-10	2	8	1
lundi	I-0.3-10	2	9	1
lundi	I-0.3-10	7	1	1
lundi	I-0.3-12	2	1	1
lundi	I-0.3-12	2	2	1

lundi	I-0.3-12	2	3	1
lundi	I-0.3-12	2	4	1
lundi	I-0.3-12	2	5	1
lundi	I-0.3-12	2	6	1
lundi	I-0.3-12	2	7	1
lundi	I-0.3-12	2	8	1
lundi	I-0.3-12	2	9	1
lundi	I-0.3-12	90	1	0
